# Effects of noninvasive brain stimulation in the treatment of poststroke depression: an overview of systematic reviews

**DOI:** 10.3389/fneur.2026.1723901

**Published:** 2026-03-25

**Authors:** Shi Liu, Zixin Huang, Jie Tan, Ning Zhao

**Affiliations:** 1 College of Acupuncture & Tuina and Rehabilitation, Hunan University of Chinese Medicine, Changsha, Hunan, China; 2 Department of Rehabilitation, Shenzhen Nanshan People’s Hospital/Affiliated Nanshan Hospital of Shenzhen University, Shenzhen, Guangdong, China

**Keywords:** AMSTAR-2, grade, noninvasive brain stimulation, overview of systematic reviews, poststroke depression, PRISMA

## Abstract

**Background:**

In recent years, although numerous meta-analyses/systematic reviews (MAs/SRs) have explored the therapeutic effect of noninvasive brain stimulation (NIBS) on poststroke depression, the reliability and quality of its clinical evidence remain uncertain. Therefore, this study aims to conduct an overview of systematic reviews to evaluate the effectiveness and safety of NIBS for PSD, thereby providing evidence-based support for clinical decision-making.

**Methods:**

A comprehensive search of multiple databases, including PubMed, EMBASE, the Cochrane Library, Web of Science, CNKI, VIP, Wan Fang, and CBM, was conducted to identify systematic reviews and meta-analyses of NIBS for PSD. Following the literature screen and data extraction, the quality of the included studies was assessed using the PRISMA statement for reporting quality, the AMSTAR-2 tool for methodological quality, and the GRADE system for evidence quality. We extracted the main outcome indicators of depressive symptoms and the secondary outcome indicators of neurological function, cognitive function, daily living ability, anxiety symptoms, clinical efficiency and adverse reactions for analysis.

**Results:**

A total of 20 MAs/SRs were included. According to the PRISMA statement, 6 (30%) reports were relatively complete, 13 (65%) had problems, and 1 (5%) had serious information deficiencies. The results of the AMSTAR-2 evaluation revealed that 3 articles (15%) were of moderate quality, 5 (25%) were of low quality, and 12 (60%) were of critically low quality. Thus, the overall quality was not high. The GRADE evaluation revealed that a total of 66 evidence bodies were included: 9 (13.6%) had moderate evidence, 15 (22.8%) had low-level evidence, and 42 (63.6%) had very low-level evidence, and no high-quality outcome indicator was identified.

**Conclusion:**

The included studies revealed that NIBS is an effective and safe treatment for PSD. However, because the methodology and results of the MAs/SRs were generally not of high quality, the reliability of the conclusions is limited to a certain extent. Future research should focus on conducting more high-quality, large-sample, multicenter follow-up studies to further verify the value of applying NIBS in PSD treatment.

**Systematic review registration:**

https://www.crd.york.ac.uk/PROSPERO/view/CRD42025633044, identifier CRD42025633044.

## 1 Introduction

Poststroke depression (PSD) is one of the significant neuropsychiatric complications of stroke. Studies have shown that PSD is not only common, with an overall incidence of 27%, but also has a rapid onset, with a cumulative incidence of 38% in 1 year (1). Most patients experience depressive symptoms within 3 months after stroke (1). The primary symptoms of this disorder include a persistent depressed mood, markedly diminished interest in daily activities, and fatigue or loss of energy (2). Scientific research has shown that PSD significantly affects brain function and can also impair cognitive network (3) and quality of life (4), such as through psychomotor retardation (5) and executive dysfunction (6). In severe cases, patients may exhibit suicidal thoughts or behaviors (7). The presence of PSD substantially hinders the rehabilitation process and decreases the efficacy of rehabilitation, leading to increased risks of disability, mortality, and stroke recurrence, as well as a reduced likelihood of patients returning to work and regaining functional independence (8, 9).

The pathophysiological basis of PSD is complex and involves multidimensional processes; thus, it cannot be reduced to a mere psychological reaction following stroke (10, 11). Terroni et al. (12) found that the volume of infarcts within the left limbic–cortical–striatal–pallidal–thalamic (LCSPT) circuit was associated with the onset of PSD, whereas Weaver et al. (13) further showed that infarcts in the right amygdala and pallidum, along with disconnections of the right prefrontal–limbic–basal ganglia–thalamic circuit, represent critical lesion sites for PSD. The prefrontal cortex (PFC) is commonly associated with functional abnormalities in individuals with depressive disorders (14, 15). Left DLPFC hypoactivity correlates more with negative emotional judgment than with perception or attention, whereas right DLPFC hyperactivity relates to attentional modulation (16). At the neurotransmitter level, the onset of PSD is related to the significant depletion of monoamine neurotransmitters such as norepinephrine (NE), serotonin (5-HT), and dopamine (DA) (17). Additionally, the accumulation of glutamate after stroke induce neuronal apoptosis through excitotoxicity (18). In terms of neuroplasticity, reduced serum levels of mature brain-derived neurotrophic factor (BDNF) exhibit a linear negative correlation with the PSD risk (19). The development of PSD is closely linked to elevated levels of proinflammatory cytokines such as IL-1β, IL-6, and TNF-*α* (20–22).

Poststroke depression treatment involves both pharmacological and nonpharmacological approaches. The commonly used pharmacological treatments include selective serotonin reuptake inhibitors (SSRIs), such as fluoxetine, paroxetine, sertraline, citalopram, and escitalopram, serotonin–norepinephrine reuptake inhibitors (SNRIs), and tricyclic antidepressants (TCAs) (23). Nonpharmacological treatments include physical therapy (24), psychotherapy (25), hyperbaric oxygen therapy (HBO) (26) and other therapies (24). Such treatments also include traditional Chinese medicine (27). Currently, the efficacy of antidepressants in the treatment of PSD is still unclear. The use of antidepressants may increase the risk of stroke recurrence (28), and contraindications still exist when antidepressants are combined with some drugs used to treat stroke. For example, the concomitant use of SSRI antidepressants and oral anticoagulants (OACs) is associated with an increased risk of bleeding (29). Furthermore, some patients exhibit poor long-term medication adherence and may experience severe adverse reactions, including sexual dysfunction, bleeding, and hyponatremia (30).

Noninvasive brain stimulation (NIBS), an emerging nonpharmacological treatment, has broad application prospects because of its noninvasiveness, good tolerability, safety, and efficacy (31). NIBS techniques include repetitive transcranial magnetic stimulation (rTMS), transcranial direct current stimulation (tDCS), transcranial alternating current stimulation (tACS), and transcranial ultrasound stimulation (TUS) (32). rTMS induces neuronal action potentials through electromagnetic induction, providing deep cortical penetration and enabling the rapid modulation of cortical and subcortical network excitability (33). High-frequency stimulation typically increases neural activity, whereas low-frequency stimulation exerts inhibitory effects (34). In contrast, tDCS modulates resting membrane potentials via sustained low-intensity electrical currents, producing more subtle neuromodulatory effects that primarily influence spontaneous cortical neuronal activity (35). Anodal stimulation elevates cortical excitability at the neuronal level whereas cathodal stimulation suppresses it (36). Beyond these two mainstream techniques, tACS and TUS represent emerging NIBS modalities. tACS employs sinusoidal alternating currents at specific frequencies to entrain neural oscillations in a frequency- and phase-dependent manner (37), thereby synchronizing neural activity in targeted brain regions to increase neural connectivity (38). TUS overcomes the limitations of electromagnetic-based approaches by utilizing focused ultrasound waves to modulate neuronal activity through mechanical mechanisms (39).

These noninvasive neuromodulation techniques are capable of targeting key brain regions implicated in PSD, including the DLPFC, thereby exerting therapeutic effects through multiple interconnected mechanisms (38, 40–42). Specifically, they are believed to restore imbalanced neurotransmitter systems (e.g., 5-HT and DA), mitigate neuroinflammatory responses, and promote synaptic plasticity (39, 43–46).

Although a substantial body of literature suggests that NIBS can effectively ameliorate PSD (47–49), Gao et al. (50) published an overview of systematic reviews on rTMS for the treatment of PSD and indicated that the methodological quality of the included studies varied considerably and that the overall evidence quality was low, thus warranting a cautious interpretation of the conclusions. Notably, NIBS encompasses not only rTMS but also other modalities, including tDCS, tACS, and TUS (32). The overall reliability of the evidence supporting these latter three techniques remains uncertain, which presents challenges for clinical decision-making in neurorehabilitation and for the refinement of NIBS treatment protocols.

We conducted an overview of systematic reviews using standardized assessment tools, including AMSTAR-2, PRISMA, and GRADE, to rigorously evaluate the methodological quality, reporting completeness, and strength of evidence of the included studies. This approach aims to provide an evidence-based, quality assessment and informed guidance for clinical practice and future research directions.

## 2 Materials and methods

### 2.1 Protocol registration

The study protocol was registered in PROSPERO on January 16, 2025 (Registration ID: CRD42025633044).

### 2.2 Inclusion criteria

#### 2.2.1 Study design

In this study, a meta-analysis or systematic review of randomized controlled trials (RCTs) investigating the efficacy of noninvasive brain stimulation in the treatment of PSD was conducted, encompassing studies published in both English and Chinese. The NIBS techniques under investigation included primarilyTMS, tDCS, tACS, and TUS.

#### 2.2.2 Study participants

This study included patients who were definitively diagnosed with poststroke depression, regardless of sex, age, ethnicity, nationality, time of onset, or the duration of the condition.

#### 2.2.3 Interventions

The experimental group received NIBS or NIBS combined with other treatments, such as antidepressants, routine treatment, acupuncture, psychological therapy, physiotherapy, rehabilitation therapy, mindfulness decompression, and music relaxation therapy. The control group received sham stimulation, other treatments, as mentioned above, or sham stimulation combined with other therapies.

#### 2.2.4 Outcome indicators

Primary outcome measures included depressive symptoms before and after treatment, which were assessed using the Hamilton Depression Scale (HAMD), and other depression scales, such as the Montgomery–Åsberg Depression Rating Scale (MADRS) and Beck Depression Inventory (BDI).

Secondary outcome measures included neurological function evaluated using the National Institutes of Health Stroke Scale (NIHSS); cognitive function assessed using the Mini-Mental State Examination (MMSE); activities of daily living measured using the Barthel Index (BI) or the Modified Barthel Index (MBI); anxiety symptoms evaluated using the Hamilton Anxiety Rating Scale (HAMA); the depression remission rate; response rate; and adverse effects.

### 2.3 Exclusion criteria

The following exclusion criteria were employed: (1) studies involving patients without a definitive diagnosis of PSD; (2) duplicate publications; (3) conference abstracts, study protocols, animal experiments, overviews of systematic reviews, umbrella reviews, etc.; (4) incomplete data or unavailable full texts; (5) studies lacking relevant outcome measures; and (6) network meta-analyses.

### 2.4 Retrieval strategy

A combination of subject words and free words was used to conduct computerized searches in both the Chinese and English databases, including PubMed, EMBASE, the Cochrane Library, Web of Science, the China Knowledge Network (CNKI), VIP, Wan Fang, and the China Biomedical Literature Database (CBM). MAs/SRs of rTMS, tDCS, tACS, and TUS for the treatment of poststroke depression were collected. The search time limit was from the establishment of the database to November 23, 2024, and the reference lists of the included studies were manually searched.

The search terms included depression, poststroke, stroke, cerebrovascular accident, transcranial magnetic stimulation, transcranial direct current stimulation, transcranial alternating current stimulation, transcranial ultrasound stimulation, noninvasive brain stimulation, meta-analysis, and systematic review. The specific search strategy for PubMed is included in Appendix A, and the strategies for the other databases are detailed in Appendix B.

### 2.5 Literature screening and data extraction

Two researchers (SL and ZXH) independently conducted literature searches and data extraction using the operational procedures outlined below. Two researchers imported the retrieved studies into EndNote software. First, duplicate studies were removed, and then the initial screen was performed by reading the title and abstract. After the irrelevant studies were excluded, the studies that may be helpful were selected. Finally, the full texts were carefully read for rescreening, and the studies that did not meet the inclusion criteria were excluded. Studies that met the inclusion criteria were included, and the data were extracted. The extracted data included (1) basic information on the included studies, such as the first author, publication year, study type, number of included studies, and total sample size; (2) intervention methods used in the experimental and control groups; (3) methodological and evidence quality assessment tools; (4) outcome measures; and (5) main conclusions. If the original data were incomplete, the author was contacted to obtain supplemental information. Finally, two researchers (SL and ZXH) cross-checked the extracted data. In cases of disagreement, a third researcher (NZ) was consulted to discuss the information and come to a consensus.

### 2.6 Evaluation methods

#### 2.6.1 Methodological quality assessment

The AMSTAR2 scale (51) contains 16 items, which are described as “yes” and “no”; some items can be evaluated as “partial yes.” Items 2, 4, 7, 9, 11, 13 and 15 are considered critical. Briefly, if no defect in a single noncritical item exists, the quality of the study is “high”; if more than one defect in noncritical items are present but no defect in a critical item exists, the study quality is “moderate”; a defect in a critical item is present with or without defects in noncritical items, the quality of the study is “low”; and if defects in more than one critical item are present with or without defects in noncritical items, the quality of the study is “critically low.”

#### 2.6.2 Assessment of reporting quality

The PRISMA2020 statement (52) includes 27 items in seven fields. Each item scored the reporting quality of the study according to the degree of conformity of the included literature reports. If it is fully compliant, it is a “complete report,” which receives 1 point; if partially compliant, it is a “partial report,” and the score is 0.5; if it is not mentioned, it is marked as “not reported,” which is scored 0 points. The total score was 27 points. Studies scoring between 22 and 27 points are considered to have relatively complete reporting, those scoring between 16 and 21 points are deemed to have some reporting deficiencies, and those scoring ≤15 points are considered to have serious reporting deficiencies (53). The PRISMA quality assessment scores of the included studies were recorded, and these data were input into Excel 3.8.0 software to construct a radar map. The interpretation of the radar map is as follows: high-quality studies cover a large area on the radar map, and the shape is more balanced and closer to the outer ring; the low-quality studies cover a smaller area, and the shape shrinks inward. The larger the area covering the radar chart, the higher the quality of the report of the systematic evaluation or meta-analysis, the better the transparency, integrity and standardization of the research process and results, and the higher the reference value. Therefore, when studies are selected, priority should be given to those with large radar map areas and balanced shapes.

#### 2.6.3 Evidence quality assessment

The GRADE system (54) evaluates the quality of evidence for outcome measures across five domains: (1) risk of bias, (2) inconsistency of the results, (3) indirectness of the evidence, (4) imprecision, and (5) publication bias. The evidence quality of the outcome indicators included in the studies was comprehensively evaluated. The quality of the evidence was divided into “high” (no downgrading), “moderate” (downgraded by one level), “low” (downgraded by two levels), or “very low” (downgraded by three or more levels).

### 2.7 Data synthesis

Two researchers (SL and ZXH) analyzed the included studies, sorted the extracted data in tabular form, integrated the research results, and conducted a descriptive analysis. For the primary and secondary outcomes of the overview, we obtained the effect estimates and 95% confidence intervals (CIs) from the MAs/SRs performed by the authors of the systematic reviews. Due to the heterogeneity between the included meta-analyses/systematic reviews, quantitative synthesis was not performed, and only a descriptive analysis was used to qualitatively analyze the results.

## 3 Results

### 3.1 Literature retrieval results

**Figure 1:**
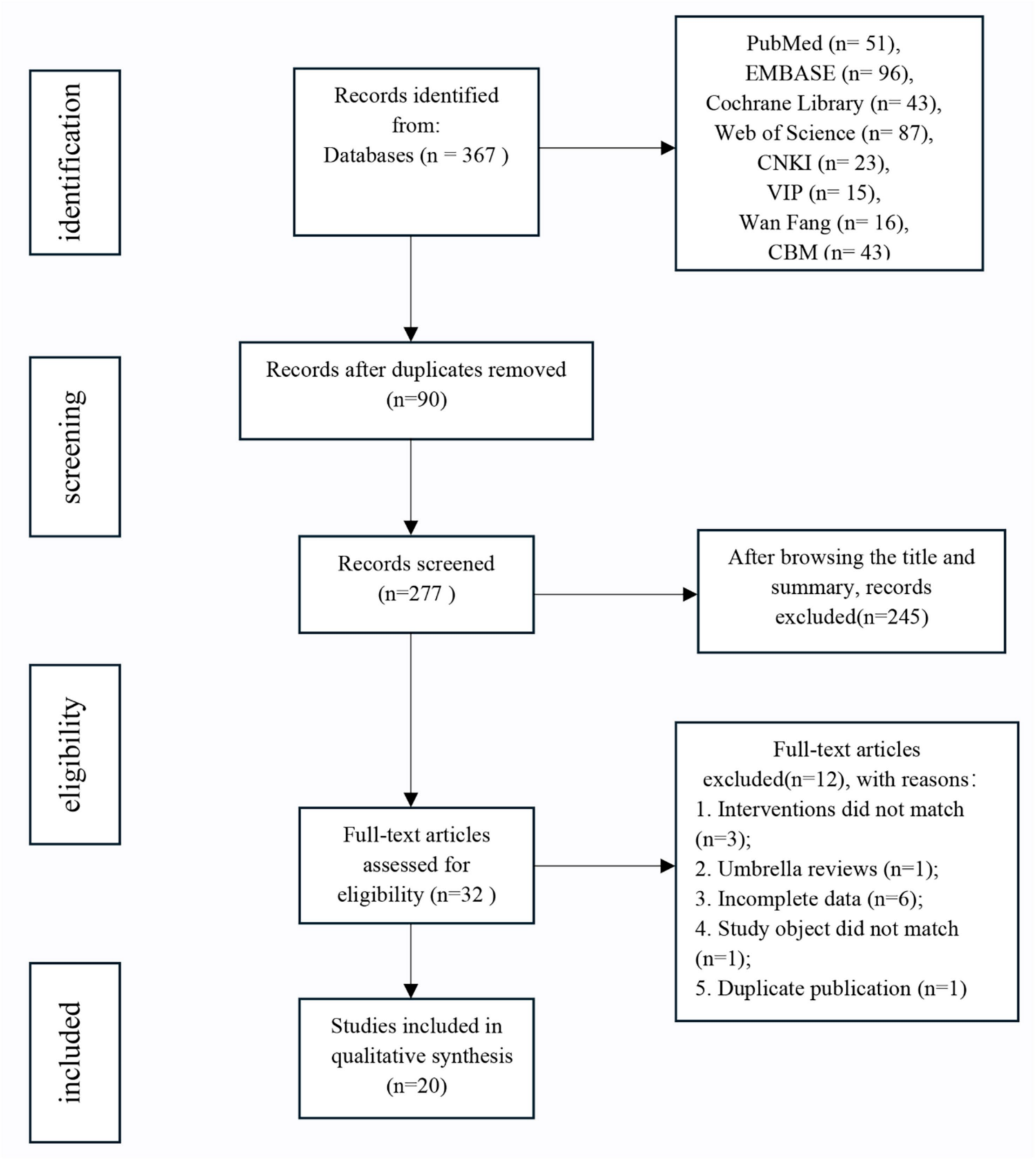
Literature screening flow chart.

Based on the search strategy, a total of 367 articles were retrieved from the following sources: PubMed, EMBASE, Cochrane Library, Web of Science, CNKI, VIP, Wan Fang, and CBM. After 90 duplicates were removed using EndNote, 245 irrelevant articles were excluded following a review of the titles and abstracts, leaving 32 articles for further evaluation. Following a full-text review, 12 articles were excluded, resulting in the final inclusion of 20 MAs/SRs (55–74). Among these studies (9 in English and 11 in Chinese), the primary intervention was rTMS, which was employed in 16 studies. NIBS and tDCS were each used in 2 studies. No studies involved tACS or TUS. The literature screening process is illustrated in Figure 1.

### 3.2 Basic characteristics of the included studies

A total of 20 articles published from the establishment of the database to November 23, 2024, were included. The number of original rTMS studies included ranged from 3 to 32, with sample sizes ranging from 81 to 2,489 patients. Moreover, the number of original tDCS studies included varied from 2 to 8, with sample sizes ranging from 138 to 412 patients. The interventions used in the experimental group were diverse and included rTMS, tDCS, or their combination with other treatments, such as antidepressants, routine treatment, acupuncture, psychological therapy, physiotherapy, rehabilitation therapy, mindfulness decompression, and music relaxation therapy. The interventions used in the control group included sham stimulation, other treatments, or sham stimulation combined with other therapies. The antidepressants used included fluoxetine, citalopram, escitalopram, mirtazapine, duloxetine, flupentixol melitracen, sertraline, zoloft, paroxetine, venlafaxine, and Chaihu shugan san. For the risk of bias assessment tools, 17 studies (56–67, 69–71, 73, 74) used the Cochrane tool, 1 study (68) used the PEDro scale, 1 study (72) employed both the Cochrane tool and the PEDro scale, and 1 study (55) utilized the CONSORT statement among the included articles. Only 1 article (67) applied the GRADE system to assess the evidence quality. In terms of conclusions, most studies suggested that rTMS and tDCS have certain advantages in treating poststroke depression. The general characteristics of the included studies are summarized in Tables 1, 2.

**Table 1:** Basic characteristics of the included rTMS studies

Authors	Country	Age (years)	Study type	Trials/n	Therapy group	Control group	Characteristics of the stimulus	Outcomes	Methodological evaluation tools	Main conclusions
Jin et al.(2016)	China	>18	Meta-analysis	24/1658	rTMS + acupuncture, rTMS + antidepressants + physical therapy (music, etc.) + traditional Chinese acupuncture + psychological therapy, rTMS + routine treatment, rTMS + ①/④/⑤/⑦, rTMS + ⑥ + psychological counseling, rTMS + ⑦ + psychological therapy, rTMS + routine treatment	Sham-rTMS + ⑥ + psychological therapy, acupuncture, routine treatment, Sham-rTMS + routine treatment, Sham-rTMS + antidepressants, ① + psychological counseling, ⑦ + psychological therapy, ①/④/⑤/⑥/⑦/⑧, antidepressants	1/10/10–15/20 Hz applied to the left DLPFC, 1 Hz applied to the right DLPFC, 0.5/10 Hz applied to the left PFC, 0.5/1 Hz applied to the bilateral PFC, 1 Hz applied to the bilateral DLPFC; 60–110% RMT; 1–8 weeks	HAMD, NIHSS, response rate	CONSORT Statement	rTMS has a positive effect on the mood of patients with PSD. Due to the limitation of the quality of the included studies, the above conclusions still need to be verified by conducting additional large-sample, multicenter and high-quality RCTs.
Li et al.(2017)	China	N	Meta-analysis	13/1033	rTMS + ①/②/③/④/⑤/⑦	①/②/③/④/⑤/⑦	0.5/1/5/10 Hz applied to the left DLPFC, 1 Hz applied to the right DLPFC, 0.5/1 Hz applied to the bilateral DLPFC, 1HZ right dorsolateral frontal cortex; 60–100% RMT	HAMD, MBI/BI, NIHSS, MMSE	Cochrane Handbook for Systematic Reviews of Interventions	rTMS combined with antidepressants may be more effective at improving the depressive symptoms of patients with PSD than antidepressants alone, and this modality improves the neurological function, cognitive level and daily living abilities of patients.
Chen et al.(2018)	China	N	Systematic review	26/1810	rTMS, rTMS + routine treatment, rTMS + ①/③/⑥ + routine treatment + rehabilitation therapy, rTMS + antidepressants + routine treatment /rehabilitation therapy, rTMS + antidepressants, rTMS + routine treatment+rehabilitation therapy, rTMS + ②/⑤/①/⑥/⑦/⑩/⑪/acupuncture + routine treatment	Sham-rTMS, routine treatment, Sham-rTMS + routine treatment, Sham-rTMS+ antidepressants + routine treatment/ rehabilitation therapy, antidepressants + routine treatment, routine treatment+ rehabilitation therapy, ①/③ + routine treatment + rehabilitation therapy, ②/⑤/①/⑥/⑦/⑩/acupuncture + routine treatment	5/10/10–15/20 Hz applied to the left DLPFC, 10 Hz applied to the left PFC, 10 Hz applied to the right DLPFC, 3 Hz applied to the left temporal–parietal cortex, 60–110% RMT; 10 days–12 weeks	HAMD, NIHSS, BI, adverse events	Cochrane risk of bias tool	High-frequency rTMS has good efficacy and good acceptability in the treatment of PSD, but attention should be given to adverse reactions such as headache. Due to the limited quantity and quality of the included studies, the above conclusions need to be verified by conducting additional high-quality studies.
Liu et al.(2018)	China	>20	Meta-analysis	18/1376	rTMS, rTMS + antidepressants/ ①/②/⑤/⑥/⑧, rTMS + routine treatment/psychological therapy/physiotherapy, rTMS + routine treatment+physiotherapy, rTMS + ②/⑤ + routine treatment/psychological therapy, rTMS + ① + psychological therapy	Sham-rTMS, ①/②/⑤/⑥/⑧, Sham-rTMS + antidepressants/ physiotherapy, routine treatment, routine treatment + psychological therapy/②/⑤, psychological therapy, ① + psychological therapy	1/3/5/10 Hz applied to the left dorsolateral frontal cortex, 1 Hz applied to the right dorsolateral frontal cortex,1HZ prefrontal cortex, 1 Hz applied to the right DLPFC, 1 Hz applied to the bilateral DLPFC, 0.5 Hz applied to the left PFC, 1/0.5 Hz applied to the bilateral PFC, 3 Hz applied to the left temporal–parietal cortex; 60–110% RMT; 1–12 weeks	HAMD, NIHSS, MBI, adverse events	Cochrane risk of bias tool	rTMS has positive effects on depression, neurological deficits, and decreased abilities of daily living in patients with PSD. Due to the quality of the included studies, the conclusions need to be verified further.
Chen et al.(2019)	China	N	Meta-analysis	21/1626	rTMS + routine treatment, rTMS + antidepressants/①/③/④/⑤/⑥/⑦/acupuncture + routine treatment, rTMS + acupuncture	routine treatment, antidepressants/①/③/④/⑤/⑥/⑦/acupuncture + routine treatment, Sham-rTMS + routine treatment, Sham-rTMS + ⑥ + routine treatment, acupuncture	1 Hz applied to the left DLPFC, 1 Hz applied to the right DLPFC, 1 Hz applied to the bilateral DLPFC, 1 Hz applied to the left PFC,0.5/1 Hz applied to the left frontal cortex, 0.5/1 Hz applied to the bilateral frontal cortex; 20–110% RMT; 1–12 weeks	HAMD, NIHSS, MMSE, BI, adverse events	Cochrane Handbook for Systematic Reviews of Interventions	Low-frequency rTMS can significantly alleviate depression in patients with PSD, improve their daily living abilities, and enhance their cognitive functions. However, the evidence for the improvement of NIHSS score with low-frequency rTMS is insufficient. Moreover, patients with PSD who are treated with low-frequency rTMS may experience mild complications such as headache. Therefore, a high-quality clinical trial is needed to validate these results.
Ding et al.(2019)	China	N	Meta-analysis	3/81	rTMS + routine treatment	Sham-rTMS + routine treatmentblank control	10 Hz applied to the left DLPFC, 10 Hz applied to the bilateral DLPFC, 1/5 Hz applied to the bilateral occipital cortex; 80–110% MT; 5 days–8 weeks	Depressive symptoms	Cochrane Handbook for Systematic Reviews of Interventions 5.1.0	rTMS can improve poststroke depression to a certain extent. However, considering that few RCTs meet the inclusion and exclusion criteria and the methodological information of the study is not perfect, multicenter and high-quality RCTs are needed to verify this conclusion in the future.
Liu et al.(2021)	China	N	Meta-analysis	25/1901	rTMS, rTMS+routine treatment/ pharmacological, rTMS+routine treatment+pharmacological, rTMS+①/②/③/④/⑤/⑨	Sham-rTMS, Sham-rTMS+③/routine treatment, routine treatment+pharmacological, pharmacological,routine treatment, ①/②/③/④/⑤/⑨	2–12 weeks	HAMD, NIHSS, MBI/BI, SDS, MMSE, response rate	Cochrane handbook for systematic reviews of interventions	The reduction of HAMD and NIHSS scores in rTMS treatment of poststroke depression, the improvement of ADL and MMSE scores and the effect of curative effect were significant.
Ma et al.(2023)	China	N	Meta-analysis	9/729	rTMS, rTMS+②	②	N	HAMD, NIHSS, BI, response rate, adverse events	Cochrane reviewer handbook quality evaluation criteria	Citalopram combined with rTMS can improve the depressive symptoms, neurological function and daily living ability of poststroke depression, and the adverse reactions are comparable.
Wang et al.(2023)	China	N	Systematic review and Meta-analysis	28/2046	rTMS+routine treatment, rTMS+routine treatment+antidepressant, rTMS+ antidepressant	Routine treatment, routine treatment+ antidepressant, Sham-rTMS+routine treatment	1 Hz left DLPFC, 0.5/1 Hz bilateral prefrontal lobes, 0.5 bilateral frontal lobes, 0.5/1 Hz right DLPFC, 0.5/1/0.5–1 Hz left frontal lobe; 1-12 weeks	HAMD, SDS, BDI, MBI/BI, MMSE, NIHSS,PHQ-9, adverse events	Cochrane Intervention Systematic Review Manual 5.1.0 Tools	Low-frequency repetitive transcranial magnetic stimulation can significantly reduce the depressive symptoms of patients with poststroke depression and can also effectively improve the recovery of neurological function to a certain extent. Low-frequency repetitive transcranial magnetic stimulation treatment has fewer adverse reactions, and the safety can be accepted.
Wang et al. (2024)	China	>20	Meta-analysis	8/542	rTMS, rTMS+①/mindfulness decompression	Sham-rTMS, Sham-rTMS+psychological therapy/mindfulness decompression, routine treatment, pharmacological, ①/③	10/20 Hz DLPFC; 60–110% RMT; 1–6 weeks	HAMD, NIHSS, HAMA, MMSE, PSQI, BDI, adverse events	Cochrane risk of bias tool Tools	Current clinical evidence shows that HF-rTMS treatment can effectively improve the depressive symptoms and activities of daily living in stroke patients, and it is safe and well tolerated. However, due to the limitations of the quantity and quality of the included studies, the above conclusions need to be verified by more high-quality studies.
Tian et al.(2011)	China	N	Meta-analysis	7/281	rTMS + conventional rehabilitative training	conventional rehabilitative training	7–30 days	HAMD, MMSE, NIHSS, line bisection, line cancellation	Cochrane Handbook for Systematic Reviews of Interventions	TMS effectively improved poststroke dysfunction, manifested by improved cognitive function and memory performance compared with controls.
Shen et al.(2017)	China	N	Systematic review and Meta-analysis	22/1764	rTMS, rTMS + regular treatment/acupuncture, rTMS + ①/④/⑤/⑦/acupuncture+regular treatment, rTMS +antidepressant	regular treatment, regular treatment + ①/④/⑤/⑦/acupuncture, Sham-rTMS, Sham-rTMS + acupuncture+routine treatment, Sham-rTMS + regular treatment/acupuncture/ antidepressant, antidepressant	1/10/10–15 Hz applied to the left DLPFC, 1/10 Hz applied to the right DLPFC, 1 Hz applied to the bilateral DLPFC, 0.5/1 Hz applied to the bilateral prefrontal cortex, 3 Hz applied to the left temporal–parietal cortices; 60–110% RMT; 7 sessions–280 sessions	HAMD, MBI/BI, NIHSS, MADRS, BDI, SDS, adverse events, response rate, remission rate,etc.	Cochrane Handbook for Systematic Reviews of Interventions; grading of recommendations, assessment, development, and evaluation	In the present meta-analysis, the positive findings suggest that rTMS has beneficial effects on PSD. However, these findings should be interpreted with caution because of heterogeneity and potential biases.
Liu et al.(2019)	China	N	Systematic review and Meta-analysis	17/1171	rTMS, rTMS + ①/④/⑨, rTMS + exercise, rTMS + routine treatment + physiotherapy/rehabilitation/①/②/④/⑥/⑦	Sham-rTMS, routine treatment, Sham-rTMS +exercise, Sham-rTMS+routine treatment, routine treatment + physiotherapy/①/②/④/⑥/⑦, ①/⑨, rehabilitation	10 Hz applied to the left DLPFC, 60–110% RMT; 2–12 weeks	HAMD, NIHSS, BI, attrition rate, adverse events, response rate, remission rate	Physiotherapy evidence database scoring system	HF-rTMS is an effective intervention for the treatment of poststroke depression, but the safety of treatment should be further verified by large sample multicenter trials.
Shao et al.(2021)	China	N	Systematic review and Meta-analysis	7/351	rTMS, rTMS + routine treatment, rTMS + routine treatment+①/⑥	Sham-rTMS, routine treatment, Sham-rTMS +⑥, routine treatment + ①	10 Hz applied to the left DLPFC, 1 Hz applied to the right DLPFC, bilateral rTMS, unilateral rTMS, 110% RMT; 1–4 weeks	HAMD, NIHSS, MMSE, MDRS, BDI, remission rate,	Cochrane Handbook for Systematic Reviews of Interventions	rTMS could be an effective treatment for patients with PSD. Further clinical studies with larger sample sizes and clearer subgroup definitions are needed to confirm these outcomes.
*Liang et al.(2022)	China	N	Systematic review and Meta-analysis	32/2489(the sample size in rTMS research)	rTMS, rTMS + ①/②/③/④/⑤/⑥/⑨/⑩	①/②/③/④/⑤/⑥/⑨/⑩	0.5/1/3/5/10/10-15/20 Hz applied to the left DLPFC, 0.5/1/5/10 Hz applied to the right DLPFC, 0.5/1 Hz applied to the bilateral DLPFC, 21 Hz applied to the parietal CZ region posterior 1 cm, 3 Hz applied to the left temporo-parietal lobe, 0.5 Hz applied to the bilateral prefrontal lobes, 1 Hz applied to the bilateral dorsolateral forehead; 80–110% MT; 1–8 weeks	HAMD, MBI, total effect rate, adverse events	Cochrane risk of bias tool	LF-rTMS (≤ 10 Hz) combined with antidepressants is more effective than antidepressants alone in the treatment of patients with PSD, and no significant adverse reactions were observed. Combination therapy with HF-rTMS (>10 Hz) showed no advantage in treating PSD.
*Shen et al.(2022)	China	N	Systematic review and Meta-analysis	7/258(the sample size in rTMS research)	rTMS	Sham-rTMS, placebo	1/10 Hz applied to the left DLPFC, 1 Hz applied to the left parietal occipital cortex, 5 Hz applied to the right parietal occipital cortex; 80–110% MT	PSD-related scales, adverse events	Cochrane risk of bias tool	rTMS was demonstrated to be an effective and safe treatment for PSD. More large-scale studies are needed to explore the effects of rTMS with different frequencies on PSD.
Wang et al.(2024)	China	N	Systematic review and Meta-analysis	10/524	rTMS, rTMS + antidepressants	Sham-rTMS	5/10/20 Hz applied to the L-DLPFC; 2–8 weeks	HAMD, PHQ-9, BDI, GDS, SDS, response rate, remission rate	Cochrane handbook for systematic reviews of interventions	High-frequency rTMS targeting the left DLPFC had significant therapeutic efficacy for PSD.
Pan et al.(2023)	China	N	Systematic review and Meta-analysis	16/1463	rTMS + ①/③/④/⑤/⑥/⑨	①/③/④/⑤/⑥/⑨	0.5/1 Hz applied to the left frontal lobe, 0.5/1 Hz applied to the right frontal lobe, 0.5/1 Hz applied to the bilateral frontal lobes, 1 Hz applied to the bilateral dorsolateral region; 60–100% RMT; 10 days–12 weeks	HAMD, NIHSS, MMSE, interleukin-6, tumor necrosis factor-α,response rate	Cochrane Handbook for Systematic Reviews of Interventions 5.3, physiotherapy evidence database scoring system	Low-frequency rTMS combined with antidepressant treatment can reduce the patient's depression and IL-6 and TNF-α levels and enhance the patient's cognitive function. Low-frequency rTMS is associated with fewer adverse reactions, demonstrating its safety. However, studies with long-term follow-ups, different intervention sites of low-frequency rTMS, and different intervention frequencies (0.5 or 1 Hz) are lacking. In the future, larger sample sizes, studies of higher quality and more RCTs are needed to verify the effectiveness of low-frequency rTMS combined with other treatments for PSD.

N, Not mentioned; HAMD, Hamilton Depression Rating Scale; NIHSS, National Institutes of Health Stroke Scale; MMSE, Mini-Mental State Examination; MADRS, Montgomery–Åsberg Depression Rating Scale; BDI, Beck Depression Inventory; PSQI, Pittsburgh Sleep Quality Index; HAMA, Hamilton Anxiety Rating Scale; BI/MBI, Barthel Index/Modified Barthel Index; SDS, Self-Rating Depression Scale; PHQ-9, Patient Health Questionnaire-9; ① fluoxetine; ② citalopram; ③ escitalopram; ④ mirtazapine; ⑤ duloxetine; ⑥ flupentixol melitracen; ⑦ sertraline; ⑧ zoloft; ⑨ paroxetine; ⑩ venlafaxine; ⑪ Chaihu shugan san. *The rTMS section of the NIBS overview (from the aforementioned article Table 2).

**Table 2:** Basic characteristics of the included tDCS/TES studies

Authors	Country	Age (years)	Study type	Trials/n	Therapy group	Control group	Stimulus feature	Outcomes	Methodology evaluation tools	Main conclusion
Sun et al.(2022)	China	≥18	Meta-analysis	6/258	tDCS, tDCS+routine treatment/ routine rehabilitation/ basic treatment /②/③	Sham-tDCS, Sham -tDCS+routine treatment/ routine rehabilitation/ basic treatment /②/③, tDCS stimulation lasted only 2 nin, and the rest of the techniques were the same as those in the intervention group	Anode: left DLPFC, Cathode: right DLPFC/ right shoulder; anode and cathode are placed at the mastoid process behind the patient's ear to stimulate the cerebellar parietal nucleus; 1.2-2mA; 20–30 min; 2–8 weeks	HAMD, HAMA, MADRS, SDS, BDI	Cochrane risk of bias tool	tDCS can improve PSD, but more high-quality studies are necessary to confirm these results.
Li et al.(2022)	China	≥18	Systematic review and Meta-analysis	8/412	tDCS, tDCS+music relaxation therapy	Sham-tDCS, normalrehabilitation	Anode: left DLPFC, Cathode: right DLPFC/ right orbitofrontal cortex/right shoulder; Anode: M1of the lesioned side, Cathode: contralesional eye/ M1of contralesional side; 1.2-2 mA; 20–30 min; 2–8 weeks	HAMD, HAMA, SADQ-H, BI/MBI, BDI, SDS	Cochrane risk of bias tool	tDCS influences the improvement of PSD, but it is not clear which stimulus program is best.
*Liang et al.(2022)	China	N	Systematic review and Meta-analysis	2/222 (the sample size in TES research)	TES+①/②	①/②	10–500 μA, cranial region ear mastoid process/earlobe; 4–5 weeks	HAMD, MBI, response rate, adverse events	Cochrane risk of bias tool	TES combined with antidepressants may be more effective than antidepressants alone, but further clinical trials are needed to verify these findings.
*Shen et al.(2022)	China	N	Systematic review and Meta-analysis	3/138 (the sample size in tDCS research)	tDCS	Sham-tDCS	Anode: left DLPFC, Cathode: right DLPFC; Anode: primary motor cortex, Cathode:contralateral eye;1-2 mA; 2–4 weeks	PSD-related scales, adverse events	Cochrane risk of bias tool	tDCS was demonstrated to be an effective and safe treatment technique for PSD. More large-scale studies are essential to explore the effects of tDCS on PSD.

N, Not mentioned; HAMD, Hamilton Depression Rating Scale; MADRS, Montgomery–Åsberg Depression Rating Scale; BDI, Beck Depression Inventory; HAMA, Hamilton Anxiety Rating Scale; BI/MBI, Barthel Index/Modified Barthel Index; SDS, Self-Rating Depression Scale; SADQ-H: Stroke Aphasic Depression Questionnaire Hospital Version; ① escitalopram; ② dalixin; ③ sertraline. *The tDCS section of the NIBS overview (from the aforementioned article Table 1).

### 3.3 Results of the PRISMA evaluation of the included studies

**Figure 2:**
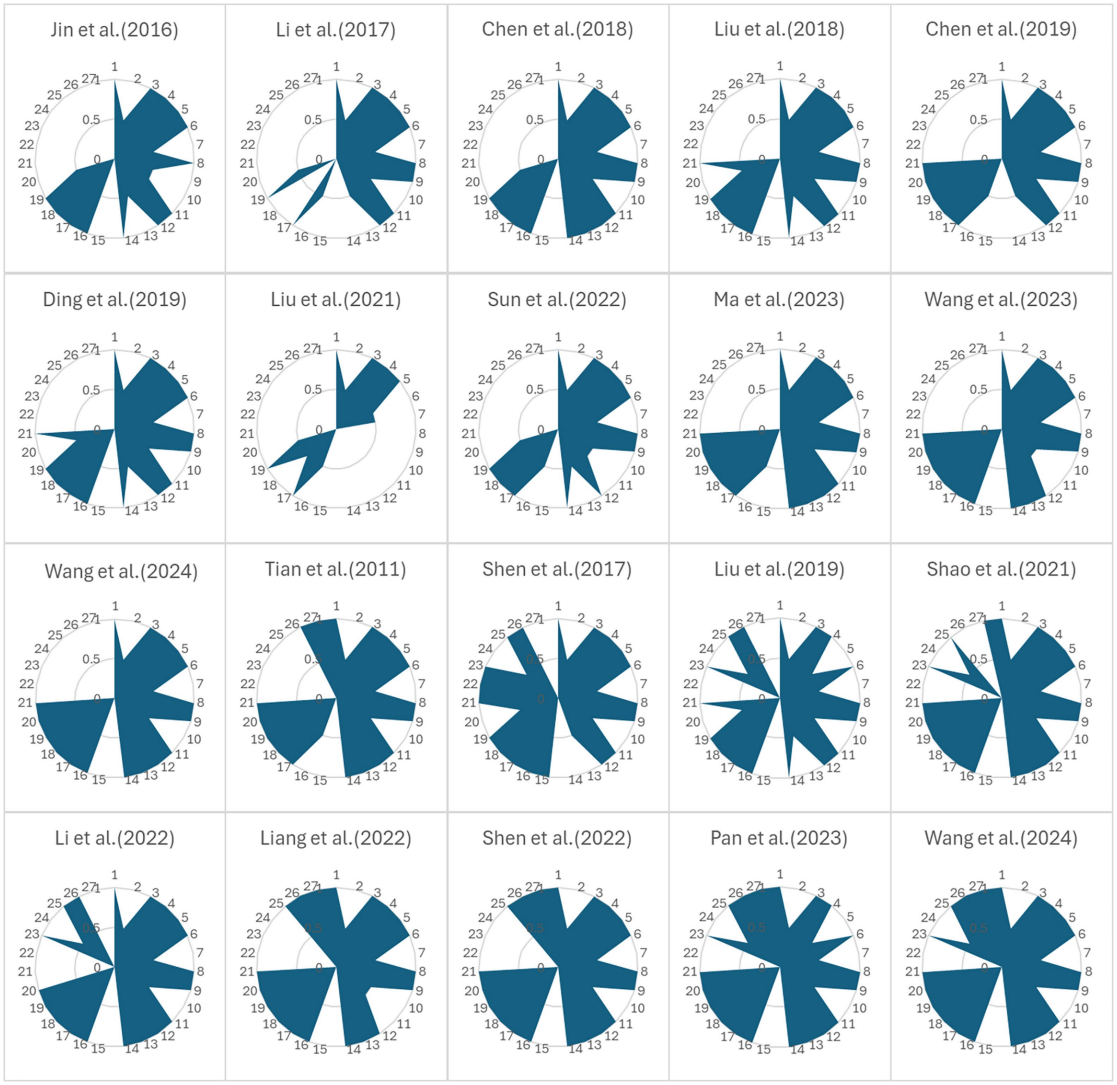
Radar charts showing the PRISMA scores for each item. Item 1, Title; item 2, Abstract; item 3, Theoretical basis; item 4, Objectives; item 5, Eligibility criteria; item 6, Information sources; item 7, Search strategy; item 8, Research selection; item 9, Data extraction; item 10, Data; item 11, Risk of bias assessment; item 12, Effect measures; item 13, Synthesis method; item 14, Reporting bias assessment; item 15, Certainty assessment; item 16, Study selection; item 17, Study characteristics; item 18, Risk of bias in studies; item 19, Results of individual studies; item 20, Synthesis of the results; item 21, Reporting bias; item 22, Certainty of evidence; item 23, Discussion; item 24, Registration and protocol; item 25, Funding; item 26, Conflicts of interest; item 27, Availability of data, code, and other materials.

In accordance with the PRISMA statement mentioned above, the PRISMA scores for the included MAs/SRs in the current study ranged from 11.5–23. Among these studies, 6 articles (67, 69, 71–74) were considered to have relatively complete reporting (22–23 points), 13 articles (55–60, 62–66, 68, 70) had some reporting deficiencies (16–21 points), and 1 article (61) had serious reporting deficiencies (11.5 points). The reasons for these reporting deficiencies were as follows: (1) the abstracts of the included studies were not sufficiently reported based on the search strategies used for each database; (2) only 1 article (67) used the GRADE system to evaluate the quality of the results; (3) the presentation of bias risks was inadequate due to missing outcomes in each synthesis; (4) only 6 articles (67, 68, 70–73) explicitly provided registration information, while the remaining articles did not report registration details or stated no registration; (5) only 8 articles (66–68, 70–74) reported conflicts of interest among the authors; 6 articles (66, 69, 71–74) reported data availability; and the remaining articles reported insufficient data. The summarized research results were visualized using radar charts generated using Excel 3.8.0 software. For details, see Figure 2. An overview of the PRISMA assessment is presented in Table 3.

**Table 3:** Overview of the PRISMA and AMSTAR2 assessments of the included MAs/SRs.

Study	PRISMA score	PRISMA category	AMSTAR2 critical flaws	AMSTAR2 rating
Jin et al.(2016)	18	problems	2	critically low
Li et al.(2017)	16	problems	3	critically low
Chen et al.(2018)	19	problems	2	critically low
Liu et al.(2018)	19.5	problems	2	critically low
Chen et al.(2019)	18.5	problems	1	low
Ding et al.(2019)	18.5	problems	2	critically low
Liu et al.(2021)	11.5	serious defects	1	low
Sun et al.(2022)	17.5	problems	2	critically low
Ma et al.(2023)	20	problems	1	low
Wang et al.(2023)	19	problems	2	critically low
Wang et al.(2024)	19.5	problems	1	low
Tian et al.(2011)	21	problems	3	critically low
Shen et al.(2017)	22	relative integrity	2	critically low
Liu et al.(2019)	20.5	problems	1	low
Shao et al.(2021)	22	relative integrity	N	moderate
Li et al.(2022)	21	problems	2	critically low
Liang et al.(2022)	22	relative integrity	2	critically low
Shen et al.(2022)	22.5	relative integrity	3	critically low
Pan et al.(2023)	22.5	relative integrity	N	moderate
Wang et al.(2024)	23	relative integrity	N	moderate

*N, No.

### 3.4 Results of the AMSTAR evaluation of the included studies

**Figure 3:**
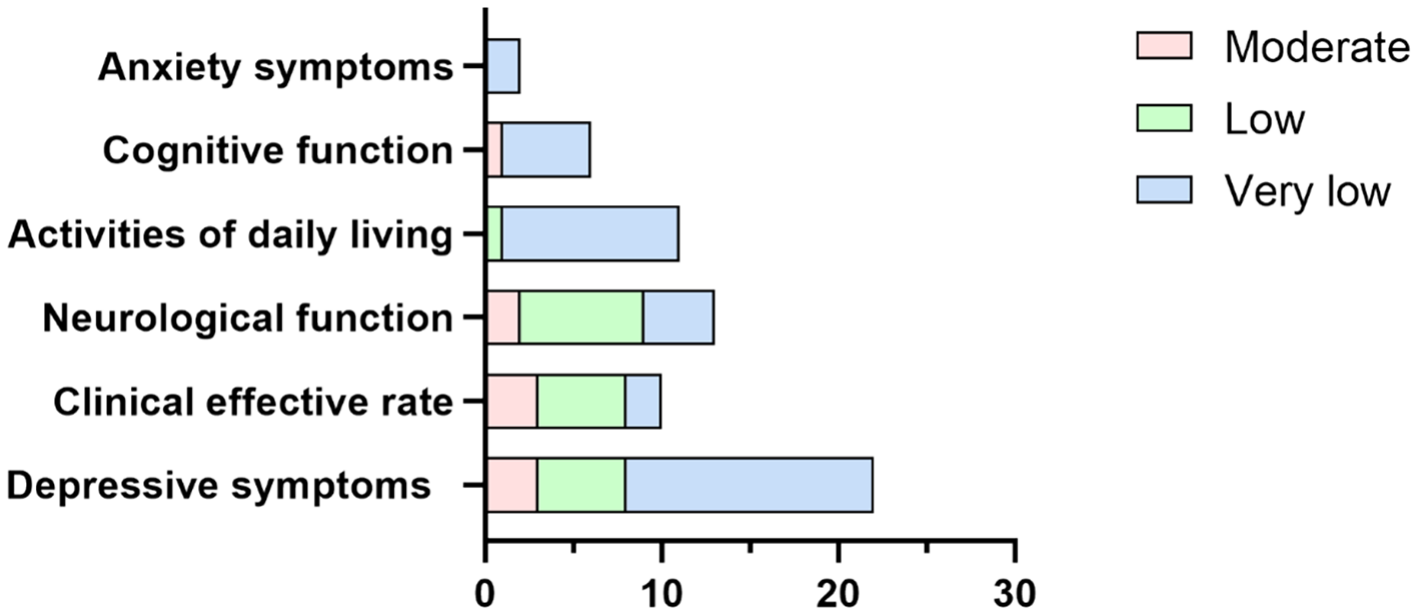
Differences in evidence quality across different outcome domains.

The AMSTAR2 scoring scale was used to evaluate the methodological quality of the 20 MAs/SRs included in this analysis. The overall methodological quality was low. Among the key items, the compliance rates were as follows: 30% for Item 2, 85% for Item 4 (partially met), 75% for Item 7 (with 15% partially met), 100% for Item 9, 100% for Item 11, 65% for Item 13, and 70% for Item 15. The primary reasons for these ratings included the following: 6 studies (67–69, 70, 72, 73) provided protocol registration with registration numbers, but none consulted domain experts during the search process, and their search strategies were incomplete; 2 studies (56, 66) failed to justify the exclusion of studies after the full-text review; 7 studies (55, 64, 66, 71, 74, 58, 67) did not discuss the critical impact of the risk of bias on the results; and 6 studies (56, 60, 62, 67, 70, 57) did not address the possibility of publication bias or its potential impact on the findings. The details are presented in Table 4. Differences in evidence quality across different outcome domains are shown in Figure 3. An overview of the AMSTAR2 assessment is presented in Table 3.

**Table 4:** AMSTAR 2 methodological quality evaluation results

Authors	Item 1	Item 2*	Item 3	Item 4*	Item 5	Item 6	Item 7*	Item 8	Item 9*	Item 10	Item 11*	Item 12	Item 13*	Item 14	Item 15*	Item 16
Jin et al.(2016)	Y	N	N	PY	Y	N	Y	PY	Y	N	Y	Y	N	N	Y	N
Li et al.(2017)	Y	N	N	PY	Y	Y	N	PY	Y	N	Y	N	Y	Y	N	N
Chen et al.(2018)	Y	N	N	PY	Y	Y	Y	PY	Y	N	Y	Y	Y	Y	N	Y
Liu et al.(2018)	Y	N	N	PY	Y	Y	Y	PY	Y	N	Y	Y	N	Y	Y	Y
Chen et al.(2019)	Y	N	N	PY	Y	Y	Y	PY	Y	N	Y	Y	Y	Y	Y	Y
Ding et al.(2019)	Y	N	Y	PY	Y	Y	Y	PY	Y	N	Y	N	Y	Y	N	N
Liu et al.(2021)	Y	N	N	PY	N	N	Y	PY	Y	N	Y	N	Y	Y	Y	Y
Sun et al.(2022)	Y	N	N	PY	Y	Y	PY	PY	Y	N	Y	Y	Y	Y	N	Y
Ma et al.(2023)	Y	N	N	PY	Y	Y	PY	PY	Y	N	Y	Y	PY	Y	Y	Y
Wang et al.(2023)	Y	N	N	PY	Y	Y	Y	PY	Y	N	Y	Y	N	Y	Y	N
Wang et al.(2024)	Y	N	N	PY	Y	Y	Y	PY	Y	N	Y	Y	Y	Y	Y	N
Tian et al.(2011)	Y	N	N	PY	Y	Y	N	Y	Y	Y	Y	Y	N	N	Y	Y
Shen et al.(2017)	Y	Y	N	PY	Y	Y	Y	PY	Y	N	Y	Y	N	Y	N	Y
Liu et al.(2019)	Y	Y	N	N	Y	Y	Y	PY	Y	N	Y	Y	Y	Y	Y	Y
Shao et al.(2021)	Y	Y	N	PY	Y	Y	Y	PY	Y	N	Y	Y	Y	Y	Y	N
Li et al.(2022)	Y	Y	N	N	Y	Y	PY	PY	Y	N	Y	Y	Y	Y	N	Y
Liang et al.(2022)	Y	N	N	PY	Y	Y	Y	PY	Y	N	Y	Y	N	Y	Y	Y
Shen et al.(2022)	Y	N	N	N	Y	Y	Y	Y	Y	N	Y	Y	N	Y	Y	Y
Pan et al.(2023)	Y	Y	N	PY	Y	Y	Y	PY	Y	N	Y	Y	Y	Y	Y	Y
Wang et al.(2024)	Y	Y	N	PY	Y	Y	Y	PY	Y	N	Y	Y	Y	Y	Y	Y

*: Key item; Y: Yes; PY: Partially; N: No; Item 1: Did the research questions and inclusion criteria for the review include the components of PICO? Item 2: Did the report of the review contain an explicit statement that the review methods were established prior to the conduct of the review, and did the report justify any significant deviations from the protocol? Item 3: Did the review authors explain their selection of the study designs for inclusion in the review? Item 4: Did the review authors use a comprehensive literature search strategy? Item 5: Did the review authors perform study selection in duplicate? Item 6: Did the review authors perform data extraction in duplicate? Item 7: Did the review authors provide a list of excluded studies and justify the exclusions? Item 8: Did the review authors describe the included studies in adequate detail? Item 9: Did the review authors use a satisfactory technique for assessing the risk of bias (RoB) in individual studies that were included in the review? Item 10: Did the review authors report on the sources of funding for the studies included in the review? Item 11: If meta-analysis was performed, did the review authors use appropriate methods for statistical combination of results? Item 12: If meta-analysis was performed, did the review authors assess the potential impact of RoB in individual studies on the results of the meta-analysis or other evidence synthesis? Item 13: Did the review authors account for RoB in individual studies when interpreting/discussing the results of the review? Item 14: Did the review authors provide a satisfactory explanation for, and discussion of, any heterogeneity observed in the results of the review? Item 15: If they performed quantitative synthesis, did the review authors carry out an adequate investigation of publication bias (small study bias) and discuss its likely impact on the results of the review? Item 16: Did the review authors report any potential sources of conflict of interest, including any funding they received for conducting the review?

### 3.5 Results of the GRADE evaluation of the included studies

The GRADE system was employed to evaluate the quality of the evidence for the outcome measures derived from the pooled effect sizes. Among the 20 included studies, six types of outcome measures were identified, encompassing 66 outcome evidence entries. The assessment revealed no high-quality evidence. The primary reasons for this result were as follows: (1) the randomized controlled trials included in the original studies exhibited deficiencies in terms of blinding, allocation concealment, and randomization methods; (2) the reliability of the results was compromised by heterogeneity in outcome measures across some studies; and (3) the limited number of included studies, small sample sizes, and asymmetry in funnel plots suggested potential publication bias, all of which undermined the accuracy of the results. The details are presented in Table 5. Differences in evidence quality across different outcome domains are shown in Figure 3.

**Table 5:** Results of the GRADE evaluation of the evidence quality

Outcomes	Authors	Number of studies	Risk of bias	Inconsistency	Indirectness	Imprecision	Publication bias	Quality of evidence	Relative effect	95% CI
Improvement in depressive symptoms	Jin et al.(2016)	24	-1a	-2c	0	0	0	Very low	SMD=-1.36	(-1.6, -1.12)
	*Li et al.(2017)	7	-1a	-1b	0	-1d	0	Very Low	WMD=-4.24	(-6.57, -1.92)
	*Li et al.(2017)	6	-1a	0	0	0	0	Moderate	WMD=-4.00	(-4.56, -3.65)
	Chen et al.(2018)	24	-1a	-2c	0	0	0	Very low	SMD=-0.59	(-0.86, -0.32)
	Liu et al.(2018)	17	-1a	-2c	0	0	0	Very low	SMD=-1.13	(-1.42, -0.84)
	Chen et al.(2019)	21	-1a	-2c	0	0	-1f	Very Low	SMD=-0.79	(-0.99, -0.58)
	Ding et al.(2019)	3	-1a	-1b	0	0	-1e	Very low	SMD=-1.45	(-2.45, -0.45)
	Liu et al.(2021)	18	-1a	-2c	0	0	0	Very low	WMD=-2.47	(-2.99, -1.96)
	Sun et al.(2022)	3	-1a	-1b	0	0	-1e	Very low	SMD=1.93	(0.98, 2.88)
	Ma et al.(2023)	9	-1a	-2c	0	0	0	Very low	MD=-3.31	(-4.37, -2.25)
	Wang et al.(2023)	24	-1a	-1b	0	0	0	low	SMD=-1.01	(-1.20, -0.82)
	Wang et al.(2024)	8	-1a	0	0	0	0	Moderate	MD=-2.72	(–3.28, -2.15)
	Tian et al.(2011)	4	-1a	0	0	-0	0	Moderate	WMD=-6.21	(-7.55, -4.87)
	Shen et al.(2017)	24	-1a	-2c	0	-1d	-1f	Very low	MD=-6.09	(-7.74, -4.45)
	Liu et al.(2019)	15	-1a	-2c	0	0	0	Very low	SMD=-1.01	(-1.36, -0.66)
	Shao et al.(2021)	7	-1a	-1b	0	0	-1e	Very low	SMD=-1.15	(-1.62, -0.69)
	Li et al.(2022)	8	-1a	-2c	0	0	-1e	Very low	SMD=1.61	(1.02, 2.19)
	Liang et al.(2022)	34	-1a	-1b	0	0	0	Low	SMD=-1.44	(-1.86, -1.03)
	Shen et al.(2022)^△^	7	-1a	-2c	0	-1d	0	Very low	SMD= 4.92	(2.69, 7.15)
	Shen et al.(2022)^△^	3	-1a	-2c	0	-1d	0	Very low	SMD= 5.30	(1.30, 9.30)
	Pan et al.(2023)	15	-1a	-2c	0	0	0	Very low	SMD=-1.01	(-1.31, -0.70)
	Wang et al.(2024)	10	-1a	-2c	0	0	0	Very low	SMD=-1.45	(-204, -0.86)
Clinical effective rate	Jin et al.(2016)	11	-1a	0	0	-1d	0	Low	OR=5.92	(3.92, 8.94)
	Chen et al.(2018)	10	-1a	-2c	0	0	0	Very low	RR=1.66	(1.28, 2.15)
	Liu et al.(2018)	5	-1a	0	0	-1d	0	Low	OR=4.08	(2.20, 7.59)
	Liu et al.(2022)	9	-1a	0	0	0	0	Moderate	RR=1.27	(1.18, 1.37)
	Ma et al.(2023)	3	-1a	0	0	-1d	-1e	Very low	OR=2.69	(1.61, 4.49)
	Shen et al.(2017)	12	-1a	0	0	-1d	0	Low	OR=3.46	(2.52, 4.76)
	Liu et al.(2019)	8	-1a	0	0	0	0	Moderate	OR=3.31	(2.25, 4.88)
	Liang et al.(2022)	17	-1a	0	0	-1d	0	Low	OR=4.33	(3.07, 6.11)
	Pan et al.(2023)	7	-1a	0	0	0	0	Moderate	OR=1.18	(0.94, 1.49)
	Wang et al.(2024)	2	-1a	0	0	-1d	-1e	Very Low	OR=8.41	(2.52,28.12)
Improvement in neurological function	Jin et al.(2016)	6	-1a	0	0	0	-1e	Low	SMD=-0.82	(-1.2, -0.44)
	Li et al.(2017)	3	-1a	0	0	0	-1e	Low	WMD=-2.23	(-3.32, -1.14)
	Chen et al.(2018)	5	-1a	0	0	0	-1e	low	SMD=-0.62	(-0.84, -0.39)
	Liu et al.(2018)	4	-1a	0	0	0	-1e	Low	SMD=-1.00	(-1.25, -0.75)
	Chen et al.(2019)	4	-1a	-1b	0	0	-1e	Very low	SMD=-0.62	(-0.84, -0.39)
	Liu et al.(2022)	8	-1a	0	0	0	0	Moderate	WMD=-1.90	(-2.26, -1.54)
	Ma et al.(2023)	4	-1a	-1b	0	0	0	Low	MD=-1.96	(-2.73, -1.19)
	Wang et al.(2023)	5	-1a	0	0	0	-1e	Low	SMD=-0.31	(-0.55, -0.07)
	Wang et al.(2024)	3	-1a	0	0	0	-1e	Low	MD=-3.31	(-4.11, -2.50)
	Tian et al.(2011)	2	-1a	-1b	0	0	-1e	Very low	WMD=-0.89	(-1.98, 0.19)
	Shen et al.(2017)	6	-1a	0	0	-1d	0	low	MD=-2.74	(-3.33,-2.15)
	Liu et al.(2019)	4	-1a	0	0	0	0	Moderate	SMD=-0.91	(-1.19, -0.63)
	Shao et al.(2021)	3	-1a	-1b	0	0	-1e	Very low	SMD=-0.67	(-1.02, -0.32)
	Pan et al.(2023)	3	-1a	0	0	0	-1e	Low	SMD=-0.67	(-0.96, -0.38)
Improvement in activities of daily living	Li et al.(2017)	6	-1a	-1b	0	-1d	-1e	Very low	WMD=--17.49	(-21.26, -13.72)
	Chen et al.(2018)	8	-1a	-2c	0	0	0	Very Low	SMD=0.50	(0.16, 0.85)
	Liu et al.(2018)	7	-1a	-2c	0	0	0	Very low	SMD=1.56	(0.80, 2.32)
	Chen et al.(2019)	11	-1a	-2c	0	0	-1e	Very low	SMD=1.38	(0.87, 1.89)
	Liu et al.(2021)	3	-1a	0	0	-1d	-1e	Very low	WMD=2.88	(0.65, 5.10)
	Ma et al.(2023)	2	-1a	-2c	0	-1d	-1e	Very low	MD=15.95	(6.14, 25.76)
	Wang et al.(2023)	14	-1a	-2c	0	0	0	Very low	SMD=1.60	(0.94, 2.26)
	Shen et al.(2017)	7	-1a	-2c	0	0	0	Very low	SMD=1.20	(0.68, 1.72)
	Liu et al.(2019)	3	-1a	-2c	0	0	0	Very low	SMD=1.09	(0.34, 1.84)
	Li et al.(2022)	4	-1a	-2c	0	0	-1e	Very low	SMD=0.82	(0.16, 1.48)
	Liang et al.(2022)	7	-1a	-2c	0	-1d	0	Very low	MD=8.29	(5.23, 11.35)
Improvement in cognitive function	Li et al.(2017)	2	-1a	0	0	0	0	Moderate	WMD=-7.00	(-7.64, -6.36)
	Chen et al.(2019)	6	-1a	-1b	0	0	-1e	Very low	SMD=0.60	(0.31, 0.90)
	Liu et al.(2021)	2	-1a	0	0	-1d	-1e	Very low	WMD=2.15	(0.70, 3.60)
	Wang et al.(2023)	7	-1a	-2c	0	0	-1e	Very low	SMD=0.88	(0.52, 1.24)
	Shen et al.(2017)	1	-1a	-1b	0	-1d	-1e	Very low	MD=-6.21	(-9.34,-3.08)
	Shao et al.(2021)	2	-1a	-2c	0	-1d	-1e	Very low	SMD=4.07	(-1.41, 9.55)
	Pan et al.(2023)	4	-1a	-2c	0	-1d	-1e	Very low	WMD=4.19	(2.11, 6.26)
Improvement in anxiety symptoms	Sun et al.(2022)	2	-1a	-2c	0	-1d	-1e	Very low	SMD=1.64	(0.04, 3.25)
	Li et al.(2022)	3	-1a	-2c	0	-1d	-1e	Very low	SMD=1.09	(-0.22, 2.40)

0, not downgraded; -1, downgraded one level; -2, downgraded two levels; MD, mean difference; SMD, standardized mean difference; WMD, weighted mean difference; OR, odds ratio; RR, relative risk; CI, confidence interval; N, not reported; *:Li et al. (2017) is the same study (with 7 reports presenting results for HF-rTMS and 6 reports presenting results for LF-rTMS). △: Shen et al. (2022) is the same study (with 7 reports presenting results for rTMS and 3 reports presenting results for tDCS). a: Did not report the risk of bias in the included studies, including randomization, allocation concealment, blinding, completeness of outcome data, or selective reporting of results. b: Included studies with 50% ≤ I^2^ <75%. c: Included studies with 75% ≤ I^2^ <100%. d: Small sample size with wide confidence intervals. e: Few included studies, potential publication bias. f: Asymmetrical funnel plot.

### 3.6 Results of the systematic evaluation of outcome measures

#### 3.6.1 Improvement in depressive symptoms

All 20 included studies utilized the HAMD score as an outcome measure. A total of 273 RCTs were included, with evidence quality ranging from moderate to very low. Only 3 studies (56, 65, 66) reported moderate-quality outcomes. Interventions included rTMS alone or combined with other treatments; and tDCS alone or combined with other treatments. The results of the included studies (55–61, 63–69, 71–74) demonstrated that, compared with control treatments, rTMS (with evidence quality ranging from moderate to very low) significantly improved the outcomes in the treatment group (*p* < 0.05), although no clear effect of the stimulation frequency on efficacy was identified. Among the rTMS protocols, HF-rTMS applied to the left DLPFC and LF-rTMS applied to the right DLPFC represent the most consistently employed approaches. The included studies (62, 70, 71, 74) showed the superior efficacy of tDCS (which has very low evidence quality) in the treatment group compared with the control group (p < 0.05). The most consistently applied tDCS protocol involves electrode placement with the anode positioned over the left DLPFC and the cathode positioned over the right DLPFC. Studies have consistently reported significant reductions in depressive symptoms following both rTMS and tDCS interventions, with improvements in depressive symptoms representing the most comprehensively documented and stable dimension of therapeutic efficacy. However, the strength of evidence supporting the efficacy of tDCS was lower than that for rTMS.

#### 3.6.2 Secondary outcome indicators

With respect to improved neurological function (14 studies, including 2 of moderate quality), 3 studies (59, 66, 69) reported no significant difference in the efficacy of rTMS compared with the control group (*p* ≥ 0.05). With respect to activities of daily living (11 studies, the evidence quality was very low), one study (70) reported no significant difference between the tDCS group and the control group (p ≥ 0.05). In terms of cognitive function (7 studies, including 1 of moderate quality), one study (69) reported no statistically significant difference between the HF-rTMS group and the control group (*p* ≥ 0.05). For anxiety symptoms (2 studies, the evidence quality was very low), one study (70) reported no significant difference after tDCS treatment (*p* ≥ 0.05). The evidence supporting improvements in secondary outcome indicators is limited and less robust. Furthermore, due to the small sample sizes and substantial heterogeneity across interventions, the consistency of these outcomes across studies remains to be confirmed through further research.

#### 3.6.3 Adverse effects

Eleven studies (57–59, 62–65, 67, 68, 71, 74) reported adverse reactions following treatment. Among these studies, 5 studies (58, 62, 65, 67, 74) employed a descriptive analysis, while 9 studies (57–59, 62, 64, 65, 67, 68, 74) reported a higher incidence of adverse reactions in the treatment group than in the control group. Four studies (57, 59, 64, 68) reported a statistically significant difference in the occurrence of headaches in the rTMS treatment group, and one study (57) reported a statistically significant difference in dizziness in the control group. The differences in the incidence rates of other adverse reactions, such as loss of appetite, local discomfort, skin irritation, dry mouth, nausea, and vomiting, were not statistically significant. These symptoms were generally mild and resolved completely after rest.

## 4 Discussion

### 4.1 Summary of the main results

The purpose of this systematic review was to summarize the evidence on the efficacy and safety of NIBS as a treatment for PSD. A total of 20 studies were included in this study, including 2 articles on NIBS, 16 articles on rTMS, and 2 articles on tDCS. A total of 312 major studies were identified, including 22,151 subjects. The results of the included studies revealed that the efficacies of rTMS and tDCS, whether used alone or in combination with other therapies, were mostly superior to the efficacy of the control treatment. Among the 66 outcome indicators, moderate-quality evidence revealed that rTMS can reduce depressive symptoms and improve neurological function in patients with PSD. The evidence for adverse reactions is limited, but most reviews suggest that rTMS and tDCS are generally well tolerated but produce mild and transient side effects. However, the methodological quality of the included reviews was generally poor, and most studies were rated as extremely low quality according to the AMSTAR2 tool. According to the PRISMA statement, the majority of the studies did not meet the ideal reporting standards. GRADE assessments did not yield high-quality evidence, and most outcomes were classified as low-quality or very low-quality.

### 4.2 Effects of NIBS on poststroke depression

NIBS can achieve the noninvasive regulation of cortical excitability in target brain regions, with rTMS and tDCS being the most widely used techniques (75). Based on the comprehensive analysis of the included studies, most rTMS protocols employed HF-rTMS (typically 10 Hz) to stimulate the left DLPFC or LF-rTMS (typically 1 Hz) to stimulate the right DLPFC. Although the optimal stimulation target has not been definitively determined, stimulation of other brain regions, such as the bilateral prefrontal cortex and left temporoparietal lobe, also exerts therapeutic effects. In contrast, tDCS generally employs a current intensity of 1–2 mA, with the anode placed over the left DLPFC and the cathode placed over the right DLPFC. A study by Li et al. (70) revealed no significant difference between the effects of stimulating the left DLPFC and stimulating the affected primary motor cortex (M1) on improving depressive symptoms, suggesting that enhancing poststroke motor function may help alleviate poststroke depression. The underlying mechanism may be related to the ability of noninvasive brain stimulation to balance three core depression-related neurocognitive networks: the salience network, default mode network, and central executive network (76). Regarding therapeutic efficacy, some studies indicate that the efficacy of HF-rTMS and LF-rTMS in improving overall depressive symptoms remains controversial (77, 78); however, they exhibit distinct characteristics of improvement across specific symptom dimensions. HF-rTMS may be a more effective treatment for core depressive symptoms such as emotional blunting and hopelessness, particularly in patients with more severe conditions, whereas LF-rTMS appears to be more advantageous in improving sleep disturbances, anxiety, and cognitive impairment, making it more suitable for patients with milder depressive symptoms (61, 77, 78). Moreover, study findings suggest that combining rTMS and tDCS may be more effective than either treatment alone (79).

### 4.3 Overall completeness and applicability of the evidence

Most RCTs have limitations in their experimental design. They often employed cross-group pharmacotherapy comparison designs, meaning that patients received both NIBS and medication concurrently rather than undergoing rTMS or tDCS alone. This approach makes the isolation of the independent therapeutic effect of NIBS difficult. Furthermore, NIBS treatment protocols varied across studies, with differences in rTMS stimulation parameters such as frequency, intensity, target area, and treatment duration. Moreover, most studies did not provide detailed parameters such as the total pulse count, intertrain interval, or specifics of sham stimulation used in control groups. In addition, the diagnosis of the depressive status primarily relied on mainstream international or domestic psychiatric classification systems, including the DSM (III-R, IV, 5), ICD-10, or CCMD-3. However, five studies (60–62, 66, 74) provided vague descriptions of diagnostic criteria. These approaches may have led to differences in the clinical characteristics of patient populations across studies. The lack of long-term follow-up data makes evaluating the durability of treatment effects difficult. Safety reporting is also inadequate, with ill-defined adverse events and incomplete documentation, hindering definitive conclusions regarding safety.

### 4.4 Quality of the evidence

At present, MAs/SRs on NIBS for PSD exhibit limitation in reporting standardization, methodological quality, and the evidence level. An evaluation using the PRISMA checklist indicated overall suboptimal reporting quality, particularly regarding the assessment of evidence credibility, as only one study (67) utilized the GRADE system. The AMSTAR2 assessment revealed that 85% of the studies were of low or critically low methodological quality, with common issues including studies not being registered, a failure to explain the study designs included in the review, incomplete literature searches, lack of reporting on conflicts of interest and funding sources, and an insufficient discussion of the bias risk or assessment of the impact of publication bias on the results of the study. Furthermore, the GRADE system indicated that the quality of evidence was moderate, low, or very low. An assessment of outcome indicators in SRs/MAs using the GRADE system revealed that evidence quality ranged from moderate to low and very low levels. This result was due mainly to flaws in the original RCTs regarding randomization, allocation concealment, and blinding, along with high heterogeneity, small sample sizes, wide confidence intervals, and insufficient statistical power to detect clinically meaningful differences. Only a small number of reviews assessed funnel plot asymmetry or conducted Egger’s test, indicating potential publication bias.

### 4.5 Strengths and shortcomings

This study included multiple key outcome measures, including depressive symptoms, anxiety symptoms, neurological function, cognitive function, activities of daily living, and adverse reactions, providing a relatively comprehensive reflection of the potential benefits of NIBS for the overall functional recovery of PSD patients. The current evidence consistently shows that rTMS and tDCS can be effective treatments that alleviate depressive symptoms in individuals with PSD, and the DLPFC has emerged as a well-supported and important therapeutic target across studies.

However, this study has several limitations. The included studies were mainly from the Chinese literature, and the research subjects were mainly from the Chinese population, which may limit the universality of the results. In addition to rTMS and tDCS, evidence from MAs/SRs on other forms of noninvasive brain stimulation, such as tACS and TUS, remains limited. The current body of evidence is insufficient to support the routine clinical application of these two modalities in the treatment of PSD. Possible reasons include the complexity of the pathological mechanisms underlying PSD, as well as the still developing nature and early-stage clinical experience with tACS and TUS. Notably, the results of this study are primarily based on the subjective evaluation of researchers, and only a qualitative synthesis was conducted. The intervention measures in the included studies are complex, the quantitative merger and analysis of the effect size could not be conducted, and a quantitative evaluation of the results cannot be provided.

## 5 Conclusion

This study summarizes the findings of a systematic analysis of NIBS as a treatment for PSD. The results indicate that NIBS exerts a certain therapeutic effect on PSD, effectively ameliorating depressive symptoms and positively affecting overall functional recovery. Among the various efficacy dimensions, improvements in depressive symptoms are the most consistently reported outcome. Intervention protocols targeting the DLPFC appear to yield relatively high effectiveness with good consistency across studies. Regarding the use of different stimulation modalities, the current evidence does not indicate a clear overall advantage of HF-rTMS over LF-rTMS, or vice versa. Additionally, the evidence is currently insufficient to conclude that tDCS is superior to rTMS in terms of efficacy, highlighting the need for further research in this area. Notably, the overall quality of currently published studies is relatively low, which, to some extent, affects the reliability of the research findings. Therefore, readers should interpret the conclusions of this study with caution.

## Data Availability

The original contributions presented in the study are included in the article/Supplementary material, further inquiries can be directed to the corresponding authors.

## References

[1] Liu L Xu M Marshall IJ Wolfe CD Wang Y O'Connell MD. Prevalence and natural history of depression after stroke: a systematic review and meta-analysis of observational studies. PLoS Med. (2023) 20:e1004200. doi: 10.1371/journal.pmed.1004200, 36976794 PMC10047522

[2] Yuan YG. Guideline for standardized diagnosis and treatment of post-stroke depression in China. Chin Med News. (2016) 31:21. doi: 10.3969/cma.j.issn.1000-8039.2016.19.027, (in Chinese)

[3] Egorova N Cumming T Shirbin C Veldsman M Werden E Brodtmann A. Lower cognitive control network connectivity in stroke participants with depressive features. Transl Psychiatry. (2018) 7:4. doi: 10.1038/s41398-017-0038-x, 29520018 PMC5843603

[4] Oni OD Olagunju AT Olisah VO Aina OF Ojini FI. Post-stroke depression: prevalence, associated factors and impact on quality of life among outpatients in a Nigerian hospital. S Afr J Psychiatry. (2018) 24:1058. doi: 10.4102/sajpsychiatry.v24i0.1058, 30263206 PMC6138133

[5] Shi Y Lenze EJ Mohr DC Lee JM Hu L Metts CL . Post-stroke depressive symptoms and cognitive performances: a network analysis. Arch Phys Med Rehabil. (2024) 105:892–900. doi: 10.1016/j.apmr.2023.10.006, 37884084 PMC11039566

[6] Terroni L Sobreiro MFM Conforto AB Adda CC Guajardo VD. Association among depression, cognitive impairment and executive dysfunction after stroke. Dement Neuropsychol. (2012) 6:152–7. doi: 10.1590/s1980-57642012dn06030007 29213789 PMC5618962

[7] Gloria MU Jonah OE Olusanjo AC Chiebuka OE Nene JJ Nwakego AU . Post-stroke depression and suicidal ideations: relationship with gender and marital status: a cross sectional study. J Prim Care Community Health. (2024) 15:21501319241233172. doi: 10.1177/21501319241233172, 38369728 PMC10878211

[8] Shewangizaw S Fekadu W Gebregzihabhier Y Mihretu A Sackley C Alem A. Impact of depression on stroke outcomes among stroke survivors: systematic review and meta-analysis. PLoS One. (2023) 18:e0294668. doi: 10.1371/journal.pone.0294668, 38039323 PMC10691726

[9] Wu QE Zhou AM Han YP Liu YM Yang Y Wang XM . Poststroke depression and risk of recurrent stroke: a meta-analysis of prospective studies. Medicine (Baltimore). (2019) 98:e17235. doi: 10.1097/md.0000000000017235, 31626084 PMC6824697

[10] Chen J Li W Por J Liu H Shen Y Cai L. Research Progress on the pathogenesis of post-stroke depression. ACS Omega. (2025) 10:47777–89. doi: 10.1021/acsomega.5c05338, 41141779 PMC12547780

[11] Gao H Sai Y Shang R Zhong X Kong L Liu J . Post stroke depression: pathogenesis and molecular mechanisms of natural product-based interventions. Front Pharmacol. (2025) 16:1595160. doi: 10.3389/fphar.2025.1595160, 40458804 PMC12127364

[12] Terroni L Amaro E Iosifescu DV Tinone G Sato JR Leite CC . Stroke lesion in cortical neural circuits and post-stroke incidence of major depressive episode: a 4-month prospective study. World J Biol Psychiatry. (2011) 12:539–48. doi: 10.3109/15622975.2011.562242, 21486107 PMC3279135

[13] Weaver NA Lim JS Schilderinck J Biessels GJ Kang Y Kim BJ . Strategic infarct locations for poststroke depressive symptoms: a lesion- and disconnection-symptom mapping study. Biol Psychiatry Cogn Neurosci Neuroimag. (2023) 8:387–96. doi: 10.1016/j.bpsc.2021.09.002, 34547548

[14] Pizzagalli DA Roberts AC. Prefrontal cortex and depression. Neuropsychopharmacology. (2022) 47:225–46. doi: 10.1038/s41386-021-01101-7, 34341498 PMC8617037

[15] Myers J Xiao J Mathura R Shofty B Pirtle V Adkinson J . Intracranial directed connectivity links subregions of the prefrontal cortex to major depression. medRxiv - Psychiatry Clin Psychol. (2024) 16:09. doi: 10.1101/2024.08.07.24311546 PMC12238457 40628743

[16] Grimm S Beck J Schuepbach D Hell D Northoff G. Imbalance between left and right dorsolateral prefrontal cortex in major depression is linked to negative emotional judgment: an fMRI study in severe major depressive disorder. Biol Psychiatry. (2008) 63:369–76. doi: 10.1016/j.biopsych.2007.05.033, 17888408

[17] Xiao-Wei JI Chun-Ling WU Wang XC Liu J Jian-Zhong BI Wang DY. Monoamine neurotransmitters and fibroblast growth factor-2 in the brains of rats with post-stroke depression. Exp Ther Med. (2014) 8:159–164. doi: 10.3892/etm.2014.1674 24944615 PMC4061212

[18] Yu SP Jiang MQ Shim SS Pourkhodadad F Wei L. Neuropharmacological insights into glutamate homeostasis in post-stroke depression regulated by astrocytes. Mol Neurodegener. (2023) 18:43. doi: 10.1186/s13024-023-00636-1 37400870 PMC10318843

[19] Chang X He Y Liu Y Fei J Qin X Song B . Serum brain derived neurotrophic factor levels and post-stroke depression in ischemic stroke patients. J Affect Disord. (2024) 361:341–7. doi: 10.1016/j.jad.2024.06.050, 38897298

[20] Zhang Y Yang Y Li H Feng Q Ge W Xu X. Investigating the potential mechanisms and therapeutic targets of inflammatory cytokines in post-stroke depression. Mol Neurobiol. (2024) 61:132–47. doi: 10.1007/s12035-023-03563-w, 37592185

[21] Feng X Ma X Li J Zhou Q Liu Y Song J . Inflammatory pathogenesis of post-stroke depression. Aging Dis. (2025) 16:209–38. doi: 10.14336/ad.2024.0203 PMC11745428 38377025

[22] Zhou Y Zhao L Tang Y Qian S. Association between serum inflammatory cytokines levels and post-stroke depression among stroke patients: a meta-analysis and systematic review. J Psychosom Res. (2025) 190:112050. doi: 10.1016/j.jpsychores.2025.112050, 39952012

[23] Wang SS Zhou XY Zhu CY. Chinese expert consensus on clinical practice of post-stroke depression. Chin J Stroke. (2016) 11:685–93. doi: 10.3969/j.issn.1673-5765.2016.08.015, (in Chinese)

[24] Zhao FY Yue YY Li L Lang SY Wang MW Du XD . Clinical practice guidelines for post-stroke depression in China. Rev Bras Psiquiatr. (2018) 40:325–34. doi: 10.1590/1516-4446-2017-2343, 29412338 PMC6899404

[25] Zhao B Yao Y Gao T. The effect of psychological intervention on the quality of life and rehabilitation outcome of stroke patients with anxiety and depression: a systematic review. Medicine (Baltimore). (2024) 103:e40439. doi: 10.1097/md.0000000000040439, 39533617 PMC11556977

[26] Guo H Ge YR Dong YB Zhao XC Su GL Wang JC. Effect of hyperbaric oxygen on post-stroke depression. World J Psychiatry. (2023) 13:226–33. doi: 10.5498/wjp.v13.i5.226, 37303936 PMC10251359

[27] Song M Zuo Z Li YN Wu J Dai O. Overview of traditional Chinese and Western medicine in the treatment of post-stroke depression. World Chin Med. (2021) 16:1638–42. doi: 10.3969/j.issn.1673-7202.2021.10.028, (in Chinese)

[28] Juang HT Chen PC Chien KL. Using antidepressants and the risk of stroke recurrence: report from a national representative cohort study. BMC Neurol. (2015) 15:86. doi: 10.1186/s12883-015-0345-x, 26045186 PMC4455315

[29] Rahman AA Platt RW Beradid S Boivin JF Rej S Renoux C. Concomitant use of selective serotonin reuptake inhibitors with Oral anticoagulants and risk of major bleeding. JAMA Netw Open. (2024) 7:e243208. doi: 10.1001/jamanetworkopen.2024.3208, 38517440 PMC10960200

[30] Wang SM Han C Bahk WM Lee SJ Patkar AA Masand PS . Addressing the side effects of contemporary antidepressant drugs: a comprehensive review. Chonnam Med J. (2018) 54:101–12. doi: 10.4068/cmj.2018.54.2.101, 29854675 PMC5972123

[31] Yu SL Jiang WW. Research progress on the effect of non-invasive brain stimulation based on neural network on post-stroke function. Chin J Phys Med Rehabil. (2020) 42:372–6. doi: 10.3760/cma.j.issn.0254-1424.2020.04.020, (in Chinese)

[32] Li KP Wu JJ Zhou ZL Xu DS Zheng MX Hua XY . Noninvasive brain stimulation for neurorehabilitation in post-stroke patients. Brain Sci. (2023) 13:13. doi: 10.3390/brainsci13030451, 36979261 PMC10046557

[33] Siebner HR Funke K Aberra AS Antal A Bestmann S Chen R . Transcranial magnetic stimulation of the brain: what is stimulated? - a consensus and critical position paper. Clin Neurophysiol. (2022) 140:59–97. doi: 10.1016/j.clinph.2022.04.022, 35738037 PMC9753778

[34] Fitzgerald PB Fountain S Daskalakis ZJ. A comprehensive review of the effects of rTMS on motor cortical excitability and inhibition. Clin Neurophysiol. (2006) 117:2584–96. doi: 10.1016/j.clinph.2006.06.712, 16890483

[35] Sudbrack-Oliveira P Razza LB Brunoni AR. Non-invasive cortical stimulation: Transcranial direct current stimulation (tDCS). Int Rev Neurobiol. (2021) 159:1–22. doi: 10.1016/bs.irn.2021.01.001 34446242

[36] Romero Lauro LJ Rosanova M Mattavelli G Convento S Pisoni A Opitz A . TDCS increases cortical excitability: direct evidence from TMS-EEG. Cortex. (2014) 58:99–111. doi: 10.1016/j.cortex.2014.05.003, 24998337

[37] Bland NS Sale MV. Current challenges: the ups and downs of tACS. Exp Brain Res. (2019) 237:3071–3088. doi: 10.1007/s00221-019-05666-0, 31620829

[38] Haller N Senner F Brunoni AR Padberg F Palm U. Gamma transcranial alternating current stimulation improves mood and cognition in patients with major depression. J Psychiatr Res. (2020) 130:31–4. doi: 10.1016/j.jpsychires.2020.07.009, 32771678

[39] Guo J Lo WLA Hu H Yan L Li L. Transcranial ultrasound stimulation applied in ischemic stroke rehabilitation: a review. Front Neurosci. (2022) 16:964060. doi: 10.3389/fnins.2022.964060, 35937889 PMC9355469

[40] Keeser D Meindl T Bor J Palm U Pogarell O Mulert C . Prefrontal transcranial direct current stimulation changes connectivity of resting-state networks during fMRI. J Neurosci. (2011) 31:15284–93. doi: 10.1523/jneurosci.0542-11.2011 22031874 PMC6703525

[41] Park C-h Kim SE Choi YS Lee J Lee SY Kang JH . Structural-functional brain network modulation using transcranial focused ultrasound stimulation: implications on the default mode network in humans. NeuroImage. (2025) 321:121540. doi: 10.1016/j.neuroimage.2025.121540, 41110649

[42] Li Y Li K Feng R Li Y Li Y Luo H . Mechanisms of repetitive transcranial magnetic stimulation on post-stroke depression: a resting-state functional magnetic resonance imaging study. Brain Topogr. (2022) 35:363–74. doi: 10.1007/s10548-022-00894-0, 35286526

[43] Sheng R Chen C Chen H Yu P. Repetitive transcranial magnetic stimulation for stroke rehabilitation: insights into the molecular and cellular mechanisms of neuroinflammation. Front Immunol. (2023) 14:1197422. doi: 10.3389/fimmu.2023.1197422, 37283739 PMC10239808

[44] Du A Huang M Wang Z Zhou H Duan H Hu S . Using low-intensity focused ultrasound to treat depression and anxiety disorders: a review of current evidence. Brain Sci. (2025) 15:1129. doi: 10.3390/brainsci15101129, 41154223 PMC12562471

[45] Oliveira C de Freitas JS Macedo IC Scarabelot VL Ströher R Transcranial direct current stimulation (tDCS) modulates biometric and inflammatory parameters and anxiety-like behavior in obese rats Neuropeptides (2019) 73 1–10 doi: 10.1016/j.npep.2018.09.006, 30446297

[46] Guo L Zhu YM Wang YJ Yang CY. Mechanisms of repetitive transcranial magnetic stimulation on post-stroke depression treatment. Prog Biochem Biophys. (2023) 50:2437–48. doi: 10.16476/j.pibb.2023.0308, (in Chinese)

[47] Sun YY Ying L Li KX Jin W. Non-invasive brain stimulation for treating post-stroke depression: a network meta-analysis. Int J Geriatr Psychiatry. (2023) 38:e5941. doi: 10.1002/gps.5941 37283525

[48] Yi YH Zhao WJ Lv SM Zhang GH Rong YH Wang X . Effectiveness of non-pharmacological therapies for treating post-stroke depression: a systematic review and network meta-analysis. Gen Hosp Psychiatry. (2024) 90:99–107. doi: 10.1016/j.genhosppsych.2024.07.011, 39084147

[49] Bucur M Papagno C. A systematic review of noninvasive brain stimulation for post-stroke depression. J Affect Disord. (2018) 238:69–78. doi: 10.1016/j.jad.2018.05.026, 29860185

[50] Gao W Xue F Yu B Yu S Zhang W Huang H. Repetitive transcranial magnetic stimulation for post-stroke depression: an overview of systematic reviews. Front Neurol. (2023) 14:930558. doi: 10.3389/fneur.2023.930558, 37006488 PMC10061017

[51] Shea BJ Reeves BC Wells G Thuku M Hamel C Moran J . AMSTAR 2: a critical appraisal tool for systematic reviews that include randomised or non-randomised studies of healthcare interventions, or both. BMJ. (2017) 358:j4008. doi: 10.1136/bmj.j4008 28935701 PMC5833365

[52] Page MJ McKenzie JE Bossuyt PM Boutron I Hoffmann TC Mulrow CD . The PRISMA 2020 statement: an updated guideline for reporting systematic reviews. BMJ. (2021) 372:n71. doi: 10.1136/bmj.n71, 33782057 PMC8005924

[53] Zhang L Gao S Wang C Li Y Yuan H Cao L . Efficacy of repetitive transcranial magnetic stimulation in post-stroke cognitive impairment: an overview of systematic reviews. Front Neurol. (2024) 15:1378731. doi: 10.3389/fneur.2024.1378731, 38715694 PMC11075487

[54] Guyatt GH Oxman AD Vist GE Kunz R Falck-Ytter Y Alonso-Coello P . GRADE: an emerging consensus on rating quality of evidence and strength of recommendations. BMJ. (2008) 336:924–6. doi: 10.1136/bmj.39489.470347.AD, 18436948 PMC2335261

[55] Jin Y Xing GQ Guo ZW Yuan Y Mu QW. The efficacy of trans-cranial magnetic stimulation for relieving post-stroke depression:a meta-analysis. Chin J Phys Med Rehabil. (2016) 38:384–93. doi: 10.3760/cma.j.issn.0254-1424.2016.05.018, (in Chinese)

[56] Li LJ Huang J Zhang N Chen Q. Effectiveness of repetitive transcranial magnetic stimulation combined with antidepressants on post-stroke depression: a meta-analysis. Chin J Mental Health. (2017) 31:274–7. doi: 10.3969/j.issn.1000-6729.2017.04.004, (in Chinese)

[57] Chen L Chen J Jin G Hu L Li XG Zhan QL. Efficacy and safety of high-frequency repetitive transcranial magnetic stimulation for post-stroke depression:a systematic review. Chin J Phys Med Rehabil. (2018) 40:441–8. doi: 10.3760/cma.j.issn.0254-1424.2018.06.010, (in Chinese)

[58] Liu CM Wang MZ Zhang GQ. Repetitive transcranial magnetic stimulation treatment of post-stroke depression: a systematic review and meta-analysis. West China Med J. (2018) 33:1287–94. doi: 10.7507/1002-0179.201805077, (in Chinese)

[59] Chen L Chen J Jin G Hu L Li XG Zhan QL. Therapeutic efficacy of low-frequency repetitive transcranial magnetic stimulation in post-stroke depression:a meta-analysis. Chin J Med Phys. (2019) 36:736–44. doi: 10.3969/j.issn.1005-202X.2019.06.023, (in Chinese)

[60] Ding XJ Yang E. Meta analysis of repetitive transcranial magnetic stimulation treating post-stroke depression. China Mod Med. (2019) 26:16–9. doi: 10.3969/j.issn.1674-4721.2019.01.006, (in Chinese)

[61] Liu XL Zeng Q Zhao YJ Li RD Chen SP Zhan XJ . Meta-analysis of repetitive transcranial magnetic stimulation in the treatment of post-stroke depression. Guide Sci Educ. (2021) 9:270–6. (in Chinese)

[62] Sun X Sun WM Dong XL Cai YX Zhang GN Yuan Q . A meta-analysis of the effect of transcranial direct current stimulation on post-stroke depression. J Int Psychia. (2022) 49:411–5. (in Chinese)

[63] Ma ZL Xue MZ. Meta-analysis of the efficacy and safety of citalopram combined with repetitive transcranial magnetic stimulation in the treatment of poststroke depression. J Med Res. (2023) 52:55–59,64. doi: 10.11969/j.issn.1673-548X.2023.09.012, (in Chinese)

[64] Wang XY. Low frequency repetitive transcranial magnetic stimulation forpost-stroke depression: systematic review and Meta-analysis. [dissertation/master’s thesis]. Changchun: Jilin University. (2023).

[65] Wang MC Li ZW. A meta-analysis of high-frequency repetitive transcranial magnetic stimulation in the treatment of post-stroke depression. Clin J Chin Med. (2024) 16:22–7. doi: 10.3969/j.issn.1674-7860.2024.09.004, (in Chinese)

[66] Tian Y Kang L Wang H Liu Z. Meta-analysis of transcranial magnetic stimulation to treat post-stroke dysfunction. Neural Regen Res. (2011) 6:1736–41.

[67] Shen XY Liu MY Cheng Y Jia C Pan XY Gou QY . Repetitive transcranial magnetic stimulation for the treatment of post-stroke depression: a systematic review and meta-analysis of randomized controlled clinical trials. J Affect Disord. (2017) 211:65–74. doi: 10.1016/j.jad.2016.12.058, 28092847

[68] Liu CM Wang MZ Liang X Xue JY Zhang GQ. Efficacy and safety of high-frequency repetitive transcranial magnetic stimulation for Poststroke depression: a systematic review and Meta-analysis. Arch Phys Med Rehabil. (2019) 100:1964–75. doi: 10.1016/j.apmr.2019.03.012, 31002813

[69] Shao D Zhao ZN Zhang YQ Zhou XY Zhao LB Dong M . Efficacy of repetitive transcranial magnetic stimulation for post-stroke depression: a systematic review and meta-analysis of randomized clinical trials. Braz J Med Biol Res. (2021) 54:e10010. doi: 10.1590/1414-431X202010010, 33470386 PMC7812912

[70] Li Y Li HP Wu MX Wang QY Zeng X. Effects of transcranial direct current stimulation for post-stroke depression: a systematic review and meta-analysis. Clin Neurophysiol. (2022) 142:1–10. doi: 10.1016/j.clinph.2022.07.369, 35914485

[71] Liang JB Feng J He JH Jiang Y Zhang HY Chen HW. Effects of noninvasive brain stimulation combined with antidepressants in patients with Poststroke depression: a systematic review and Meta-analysis. Front Pharmacol. (2022) 13:887115. doi: 10.3389/fphar.2022.887115, 35662704 PMC9160966

[72] Pan JH Li HP Wang YS Lu L Wang Y Zhao TY . Effects of low-frequency rTMS combined with antidepressants on depression in patients with post-stroke depression: a systematic review and meta-analysis. Front Neurol. (2023) 14:1168333. doi: 10.3389/fneur.2023.1168333, 37273720 PMC10235791

[73] Wang TT Liu XT Wu XM Fan YZ Lv YT Chen B. High-frequency rTMS of the left dorsolateral prefrontal cortex for post-stroke depression: a systematic review and meta-analysis. Clin Neurophysiol. (2024) 157:130–41. doi: 10.1016/j.clinph.2023.11.019, 38103393

[74] Shen YT Cai ZY Liu FR Zhang ZH Ni GX. Repetitive transcranial magnetic stimulation and transcranial direct current stimulation as treatment of Poststroke depression a systematic review and Meta-analysis. Neurologist. (2022) 27:177–82. doi: 10.1097/NRL.0000000000000416, 35184118

[75] Kesikburun S. Non-invasive brain stimulation in rehabilitation. Turk J Phys Med Rehabil. (2022) 68:1–8. doi: 10.5606/tftrd.2022.10608, 35949977 PMC9305642

[76] Xiao Y Dong S Pan C Guo H Tang L Zhang X . Effectiveness of non-invasive brain stimulation on depressive symptoms targeting prefrontal cortex in functional magnetic resonance imaging studies: a combined systematic review and meta-analysis. Psychoradiology. (2024) 4:4. doi: 10.1093/psyrad/kkae025, 39659696 PMC11629992

[77] Yan TT He ML Gu ZT. A randomized, controlled study of high- and low-frequency rTMS on the treatment outcome for post stroke depression. Qingdao Med J. (2010) 42:81–5. doi: 10.3969/j.issn.1006-5571.2010.02.001

[78] Bo ZH Du J Wang YC. To Observe the effect of repeated transcranial magnetic stimulation with different modes on the patients with post-stroke depression and the influence of serum related indexes. Adv Chin Med. (2024) 14:1045–51. doi: 10.12677/acm.2024.1482319

[79] Zhou D Li X Wei S Yu C Wang D Li Y . Transcranial direct current stimulation combined with repetitive transcranial magnetic stimulation for depression: a randomized clinical trial. JAMA Netw Open. (2024) 7:e2444306. doi: 10.1001/jamanetworkopen.2024.44306, 39535797 PMC11561687

