# Supplementary material for: Effects of noninvasive brain stimulation in the treatment of poststroke depression: an overview of systematic reviews
Source: Front Neurol. 2026 Mar 25;17:1723901. doi: 10.3389/fneur.2026.1723901 (PMC13056648; doi:10.3389/fneur.2026.1723901)
Supplement: Supplementary file 1 [file Supplementary_file_1.docx]

**Appendix A:**

**PubMed**

| #1 | "Transcranial Magnetic Stimulation"[Mesh] |
| --- | --- |
| #2 | "Transcranial Direct Current Stimulation"[Mesh] |
| #3 | Transcranial Magnetic Stimulation[Title/Abstract] OR Transcranial Magnetic Stimulation*[Title/Abstract] OR repetitive transcranial magnetic stimulation[Title/Abstract] OR TMS[Title/Abstract] OR rTMS[Title/Abstract] |
| #4 | transcranial direct current Stimulation[Title/Abstract] OR tDCS[Title/Abstract] OR rtDCS[Title/Abstract] OR tES[Title/Abstract] OR Repetitive transcranial direct current Stimulation[Title/Abstract] OR Transcranial Electrical Stimulation[Title/Abstract] |
| #5 | transcranial alternating current stimulation[Title/Abstract] OR tACS[Title/Abstract] |
| #6 | transcranial ultrasound stimulation[Title/Abstract] OR TUS [Title/Abstract] |
| #7 | Non-invasive Brain Stimulation[Title/Abstract] OR NIBS[Title/Abstract] |
| #8 | #1 OR #2 OR #3 OR #4 OR #5 OR #6 OR #7 |
| #9 | "Stroke"[Mesh] |
| #10 | Cerebrovascular Accident[Title/Abstract] OR Brain Ischemia[Title/Abstract] OR cerebral hemorrhage[Title/Abstract] OR Stroke[Title/Abstract] OR Brain Infarction[Title/Abstract] OR Cerebral Stroke[Title/Abstract] OR Post-stroke[Title/Abstract] OR post stroke[Title/Abstract] |
| #11 | #9 OR #10 |
| #12 | "Depression"[Mesh] |
| #13 | "Depressive Disorder"[Mesh] |
| #14 | Depression[Title/Abstract] OR Depressive Disorder[Title/Abstract] OR Dysthymic Disorder[Title/Abstract] OR Depressive[Title/Abstract] OR Dysthymia[Title/Abstract] OR Depressive Symptoms[Title/Abstract] OR Emotional Depression[Title/Abstract] OR post-stroke depression[Title/Abstract] |
| #15 | #12 OR #13 OR #14 |
| #16 | "Meta-Analysis as Topic"[Mesh] |
| #17 | "Systematic Reviews as Topic"[Mesh] |
| #18 | meta- analysis[Publication Type] OR Systematic Review[Publication Type] OR meta- analysis[Title/Abstract] OR meta- analyses[Title/Abstract] OR meta[Title/Abstract] OR meta reviews[Title/Abstract] OR system* review[Title/Abstract] OR system* overview[Title/Abstract] OR system* assessment[Title/Abstract] OR system* evaluation[Title/Abstract] OR evidence synthesis[Title/Abstract] OR review literature[Title/Abstract] OR data pooling[Title/Abstract] OR clinical trial overview[Title/Abstract] |
| #19 | #16 OR #17 OR #18 |
| #20 | #8 AND #11 AND #15 AND #19 |

**Appendix B:**

**Embase**

| #1 | 'transcranial magnetic stimulation'/exp |
| --- | --- |
| #2 | 'transcranial direct current stimulation'/exp |
| #3 | 'transcranial magnetic stimulation':ab,ti OR 'repetitive transcranial magnetic stimulation':ab,ti OR 'tms':ab,ti OR 'rtms':ab,ti OR 'transcranial direct current stimulation':ab,ti OR 'tdcs':ab,ti OR 'rtdcs':ab,ti OR 'tes':ab,ti OR 'repetitive transcranial direct current stimulation':ab,ti OR 'transcranial electrical stimulation':ab,ti OR 'transcranial alternating current stimulation':ab,ti OR 'tacs':ab,ti OR ' transcranial ultrasound stimulation ':ab,ti OR ' transcranial ultrasonic brain stimulation ':ab,ti OR 'tus':ab,ti OR 'non-invasive brain stimulation':ab,ti OR 'nibs':ab,ti |
| #4 | #1 OR #2 OR #3 |
| #5 | cerebrovascular accident'/exp |
| #6 | 'cerebrovascular accident':ab,ti OR 'brain ischemia':ab,ti OR 'cerebral hemorrhage':ab,ti OR 'stroke':ab,ti OR 'brain infarction':ab,ti OR 'cerebral stroke':ab,ti OR 'post-stroke':ab,ti |
| #7 | #5 OR #6 |
| #8 | 'depression'/exp |
| #9 | 'dysthymia'/exp |
| #10 | 'depression':ab,ti OR 'depressive disder':ab,ti OR 'dysthymic disder':ab,ti OR 'depressive':ab,ti OR 'dysthymia':ab,ti OR 'depressive symptoms':ab,ti OR 'emotional depression':ab,ti |
| #11 | #8 OR #9 OR #10 |
| #12 | systematic review'/exp |
| #13 | 'meta analysis'/exp |
| #14 | 'meta- analysis':ab,ti OR 'meta- analyses':ab,ti OR 'meta':ab,ti OR 'meta reviews':ab,ti OR 'systematic review':ab,ti OR 'system review':ab,ti OR 'systematic overview':ab,ti |
| #15 | #12 OR #13 OR #14 |
| #16 | #4 AND #7 AND #11 AND #15 |

**Cochrane Library**

| #1 | MeSH descriptor: [Transcranial Magnetic Stimulation] explode all trees |
| --- | --- |
| #2 | MeSH descriptor: [Transcranial Direct Current Stimulation] explode all trees |
| #3 | (Transcranial Magnetic Stimulation ):ti,ab,kw OR (repetitive transcranial magnetic stimulation ):ti,ab,kw OR (TMS):ti,ab,kw OR (rTMS ):ti,ab,kw OR (transcranial direct current Stimulation ):ti,ab,kw OR (tDCS ):ti,ab,kw OR (rtDCS ):ti,ab,kw OR (tES ):ti,ab,kw OR (Repetitive transcranial direct current Stimulation ):ti,ab,kw OR (Transcranial Electrical Stimulation):ti,ab,kw OR (transcranial alternating current stimulation ):ti,ab,kw OR (tACS):ti,ab,kw OR  (transcranial ultrasound stimulation):ti,ab,kw OR (transcranial ultrasonic brain stimulation):ti,ab,kw OR (TUS):ti,ab,kw OR  (Non-invasive Brain Stimulation):ti,ab,kw OR (NIBS):ti,ab,kw |
| #4 | #1 OR #2 OR #3 |
| #5 | MeSH descriptor: [Stroke] explode all trees |
| #6 | (Cerebrovascular Accident ):ti,ab,kw OR (Brain Ischemia ):ti,ab,kw OR (cerebral hemrhage ):ti,ab,kw OR (Stroke):ti,ab,kw OR (Brain Infarction ):ti,ab,kw OR (Cerebral Stroke ):ti,ab,kw OR (Post-stroke ):ti,ab,kw OR (post stroke):ti, ab,kw |
| #7 | #5 OR #6 |
| #8 | MeSH descriptor: [Depression] explode all trees |
| #9 | Depression ):ti,ab,kw OR(Depressive Disder ):ti,ab,kw OR (Dysthymic Disder ):ti,ab,kw OR (Depressive ):ti,ab,kw OR (Dysthymia ):ti,ab, kw OR (Depressive Symptoms ):ti,ab,kw OR(Emotional Depression):ti,ab, kw |
| #10 | #8 OR #9 |
| #11 | (meta analysis):ab,ti, kw OR (meta analyses):ab,ti, kw OR (meta-Analysis):ab,ti, kw OR (systematic review):ab,ti,kw OR (systematic reviews):ab,ti,kw OR (systematic assessment ):ti,ab,kw OR (system assessment):ti,ab,kw OR (system evaluation ):ti,ab,kw OR (systematic evaluation ):ti,ab, kw OR (evidence synthesis ):ti,ab,kw OR (data pooling ):ti,ab, kw OR (review literature):ti,ab,kw OR (clinical trial overview):ti,ab,kw |
| #12 | #4 AND #7 AND #10 AND #11 |

**Web of Science**

| #1 | ts=(Transcranial Magnetic Stimulation OR repetitive transcranial magnetic stimulation OR TMS OR rTMS OR transcranial direct current Stimulation OR tDCS OR rtDCS OR tES OR Repetitive transcranial direct current Stimulation OR Transcranial Electrical Stimulation OR transcranial alternating current stimulation OR tACS OR transcranial ultrasound stimulation OR transcranial ultrasonic brain stimulation OR TUS OR Non-invasive Brain Stimulation OR NIBS) |
| --- | --- |
| #2 | ts=(Cerebrovascular Accident OR Brain Ischemia OR cerebral hemrhage OR Stroke OR Brain Infarction OR Cerebral Stroke OR Post-stroke ) |
| #3 | TS=(Depression OR Depressive Disorder OR Dysthymic Disorder OR Depressive OR Dysthymia OR Depressive Symptoms OR Emotional Depression ) |
| #4 | TS=(meta- analysis OR meta- analyses OR meta OR meta reviews OR Systematic Review OR system review OR systematic overview ) |
| #5 | #1 AND #2 AND #3 AND #4 |

**CBM**

| #1 | "经颅磁刺激"[不加权:扩展] |
| --- | --- |
| #2 | 经颅直流电刺激"[不加权:扩展] |
| #3 | "经颅磁刺激"[常用字段:智能] OR "TMS"[常用字段:智能] OR "重复经颅磁刺激"[常用字段:智能] OR "经颅直流电刺激"[常用字段:智能] OR "tDCS"[常用字段:智能] OR "经颅电刺激"[常用字段:智能] OR "经颅交流电刺激"[常用字段:智能] OR "tACS"[常用字段:智能] OR "经颅超声刺激"[常用字段:智能] OR "TUS"[常用字段:智能] OR "非侵入性脑刺激"[常用字段:智能] OR "无创性脑刺激技术"[常用字段:智能] |
| #4 | #1 OR #2 OR #3 |
| #5 | "卒中"[不加权:扩展] |
| #6 | "中风"[不加权:扩展] |
| #7 | "脑出血"[不加权:扩展] |
| #8 | "脑梗死"[不加权:扩展] |
| #9 | "脑梗死"[不加权:扩展] |
| #10 | "中风"[常用字段:智能] OR "卒中"[常用字段:智能] OR "脑卒中"[常用字段:智能] OR "脑血管意外"[常用字段:智能] OR "脑血管中风"[常用字段:智能] OR "脑血管障碍"[常用字段:智能] OR "脑梗塞"[常用字段:智能] OR "脑梗死"[常用字段:智能] OR "脑出血"[常用字段:智能] OR "脑溢血"[常用字段:智能] OR "缺血性中风"[常用字段:智能] OR "出血性中风"[常用字段:智能] OR "中风后"[常用字段:智能] |
| #11 | #4 OR #5 OR #6 OR #7 OR #8 OR #9 |
| #12 | "抑郁"[不加权:扩展] |
| #13 | "抑郁症"[不加权:扩展] |
| #14 | "抑郁"[常用字段:智能] OR "抑郁症"[常用字段:智能] OR "抑郁症状"[常用字段:智能] OR "抑郁情绪"[常用字段:智能] OR "抑郁状态"[常用字段:智能] OR "抑郁综合症"[常用字段:智能] OR "恶劣心境"[常用字段:智能] OR "情感障碍"[常用字段:智能] OR "精神障碍"[常用字段:智能] OR "情绪障碍"[常用字段:智能]) OR 卒中后抑郁"[常用字段:智能]) |
| #15 | #11 OR #12 OR #13 |
| #16 | "Meta分析"[不加权:扩展] |
| #17 | "系统综述"[不加权:扩展] |
| #18 | "meta分析"[常用字段:智能] OR "元分析"[常用字段:智能] OR "荟萃分析"[常用字段:智能] OR "系统评价"[常用字段:智能] OR "系统综述"[常用字段:智能]) |
| #19 | #15 OR #16 OR #17 |
| #20 | #4 AND #11 AND #15 AND #19 |

**CNKI**

（主题：中风 + 卒中 + 脑卒中 + 脑血管意外 + 脑血管中风 + 脑血管障碍 + 脑梗塞 + 脑梗死 + 脑出血 + 脑溢血 + 缺血性中风 + 出血性中风 + 中风后(精确)）AND（主题：抑郁 + 抑郁症 + 抑郁症状 + 抑郁情绪 + 抑郁状态 + 抑郁综合症 + 恶劣心境 + 情感障碍 + 精神障碍 + 情绪障碍 + 卒中后抑郁(精确)）AND（主题：经颅磁刺激 + TMS + 重复经颅磁刺激 + 经颅直流电刺激 + tDCS + 经颅电刺激 + 经颅交流电刺激 + tACS + 经颅超声刺激+ TUS + 非侵入性脑刺激 + 无创性脑刺激技术(精确)）AND（主题：系统评价 + 系统综述 + meta分析 + 元分析 + 荟萃分析(精确)）

Wang fan

**（中英文扩展&主题词扩展）：**题名或关键词:(中风 OR 卒中 OR 脑卒中 OR 脑血管意外 OR 脑血管中风 OR 脑血管障碍 OR 脑梗塞 OR 脑梗死 OR 脑出血 OR 脑溢血 OR 缺血性中风 OR 出血性中风 OR 中风后) and 题名或关键词:(抑郁 OR 抑郁症 OR 抑郁症状 OR 抑郁情绪 OR 抑郁状态 OR 抑郁综合症 OR 恶劣心境 OR 情感障碍 OR 精神障碍 OR 情绪障碍 OR 卒中后抑郁) and 题名或关键词:(经颅磁刺激 OR 重复经颅磁刺激 OR 经颅直流电刺激 OR 经颅电刺激 OR 经颅交流电刺激 OR 经颅超声刺激 OR 非侵入性脑刺激 OR 无创性脑刺激技术 ) and 题名或关键词:(系统评价 OR 系统综述 OR meta分析 OR 元分析 OR 荟萃分析)

VIP

((((((((((((题名或关键词=经颅磁刺激 OR 题名或关键词=TMS) OR 题名或关键词=重复经颅磁刺激) OR 题名或关键词=经颅直流电刺激) OR 题名或关键词=tDCS) OR 题名或关键词=经颅电刺激) OR 题名或关键词=经颅交流电刺激) OR 题名或关键词=tACS) OR 题名或关键词=经颅超声刺激) OR 题名或关键词=TUS) OR题名或关键词=非侵入性脑刺激) OR 题名或关键词=无创性脑刺激技术) AND ((((((((((((题名或关键词=中风 OR 题名或关键词=卒中) OR 题名或关键词=脑卒中) OR 题名或关键词=脑血管意外) OR 题名或关键词=脑血管中风) OR 题名或关键词=脑血管障碍) OR 题名或关键词=脑梗塞) OR 题名或关键词=脑梗死) OR 题名或关键词=脑出血) OR 题名或关键词=脑溢血) OR 题名或关键词=缺血性中风) OR 题名或关键词=出血性中风) OR 题名或关键词=中风后)) AND (((((((((题名或关键词=抑郁 OR 题名或关键词=抑郁症) OR 题名或关键词=抑郁症状) OR 题名或关键词=抑郁情绪) OR 题名或关键词=抑郁状态) OR 题名或关键词=抑郁综合症) OR 题名或关键词=恶劣心境) OR 题名或关键词=情感障碍) OR 题名或关键词=精神障碍) OR 题名或关键词=情绪障碍)) AND ((((题名或关键词=系统评价 OR 题名或关键词=系统综述) OR 题名或关键词=meta分析) OR 题名或关键词=元分析) OR 题名或关键词=荟萃分析))
